# CRISPLD2 Attenuates Intervertebral Disc Degeneration by Suppressing Oxidative Stress‐Induced Ferroptosis through the miR‐548I‐IL17A Axis

**DOI:** 10.1002/advs.202516477

**Published:** 2026-01-09

**Authors:** Yangyang Shi, Fudong Li, Tianyi Zhao, Kaiqiang Sun, Danying Zhang, Chen Yan, Ximing Xu, Jingchuang Sun, Xulin Hu, Xin Yong, Jiangang Shi, Yuan Wang

**Affiliations:** ^1^ Department of Orthopedics & Spine Surgery Laboratory of Spinal and Spinal Cord Injury Regeneration and Repair The First Affiliated Hospital of Anhui Medical University Hefei P. R. China; ^2^ Department of Orthopedic Surgery Changzheng Hospital Naval Medical University Shanghai P. R. China; ^3^ Department of Emergency and Critical Care Shanghai Changzheng Hospital Shanghai P. R. China; ^4^ Clinical Medical College and Affiliated Hospital of Chengdu University Chengdu University Chengdu P. R. China; ^5^ Key Laboratory of Birth Defects and Related Diseases of Women and Children Department of Paediatrics West China Second University Hospital Sichuan University Chengdu China

**Keywords:** CRISPLD2, ferroptosis, intervertebral disc degeneration, nucleus pulposus cells, miRNA

## Abstract

Intervertebral disc degeneration (IVDD) is a major cause of chronic low back pain, yet its molecular mechanisms remain unclear. We identified CRISPLD2 (cysteine‐rich secretory protein LCCL domain‐containing 2) as a critical regulator of nucleus pulposus cells (NPCs) homeostasis during IVDD. Single‐cell transcriptomic analysis (scRNA‐seq) revealed degenerative NPCs subsets enriched in iron metabolism and lipid peroxidation. CRISPLD2 knockdown in NPCs led to disrupted redox balance, elevated lipid peroxides, and excessive iron accumulation, promoting oxidative stress‐induced ferroptosis and disc degeneration. Mechanistically, we identified a CRISPLD2‐miR‐548I‐IL17A axis that governs ferroptotic cell death in IVDD, where CRISPLD2 deficiency was associated with reduced miR‐548I expression, accompanied by subsequent upregulation of IL17A, thereby amplifying inflammatory and oxidative stress responses. In vivo, CRISPLD2 knockdown induced spontaneous IVDD and increased pain sensitivity, while restoration of CRISPLD2 or inhibition of IL17A alleviated ferroptosis and improved NPCs survival. Adeno‐associated virus (AAV)‐mediated overexpression of CRISPLD2 successfully alleviated IVDD in lumbar spine instability and needle‐puncture models, reducing oxidative stress‐induced ferroptosis and restoring disc integrity. These findings highlight the critical role of CRISPLD2 in regulating oxidative stress‐induced ferroptosis in IVDD and suggest that targeting the CRISPLD2‐miR‐548I‐IL17A axis may provide a novel therapeutic strategy for preventing disc degeneration and alleviating discogenic pain.

## Graphical abstract

This study identifies CRISPLD2 as a key protector against IVDD. By regulating ferroptosis through the CRISPLD2–miR‐548I–IL17A axis, CRISPLD2 maintains NPCs homeostasis and reduces oxidative stress. Restoring CRISPLD2 expression effectively alleviates disc degeneration and highlights a promising therapeutic strategy for discogenic low back pain.

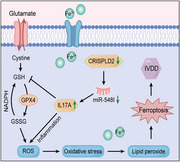

## 1 Introduction

Intervertebral disc degeneration (IVDD), a leading cause of low back pain (LBP), poses a major global health challenge by severely compromising quality of life and imposing a substantial socioeconomic burden [1, 2]. The pathogenesis of IVDD is multifactorial and involves complex interactions between mechanical, biological, and genetic factors [3, 4]. Among these, dysregulated inflammation has been recognized as a pivotal driver. In the early stages of IVDD, external insults, such as mechanical overload or trauma, and internal risk factors, including aging, genetic susceptibility, and metabolic disorders, stimulate nucleus pulposus cells (NPCs) to secrete pro‐inflammatory mediators, including prostaglandin‐endoperoxide synthase 2 (PGE2), interleukin 1 (IL‐1), interleukin 6 (IL‐6), and tumor necrosis factor‐α (TNF‐α), thereby establishing a chronic inflammatory microenvironment [5]. This inflammatory milieu leads to a disruption of extracellular matrix (ECM) homeostasis, marked by decreased expression of anabolic components (e.g., aggrecan, collagen type II, chondroitin sulfate synthase) and upregulation of catabolic enzymes such as matrix metalloproteinases (MMPs) and a disintegrin and metalloproteinase with thrombospondin motifs (ADAMTSs) [6]. Hence, targeting key regulators of inflammation to restore ECM balance holds potential as a promising therapeutic strategy for IVDD.

Cysteine‐rich secretory protein LCCL domain‐containing 2 (CRISPLD2) is an immunomodulatory protein known for its anti‐inflammatory properties and involvement in various physiological processes, including neuroregulation, programmed cell death, and metabolic homeostasis [7]. Structurally, CRISPLD2 contains a conserved LCCL domain that participates in the regulation of Toll‐like receptor 4 (TLR4)–mediated signaling pathways, including transforming growth factor‐β (TGF‐β) and nuclear factor κB (NF‐κB), ultimately suppressing inflammatory cascades [8]. Previous studies have demonstrated that CRISPLD2 expression is upregulated by high mobility group box 1 (HMGB1) in a murine model of sepsis, and that CRISPLD2 in turn attenuates HMGB1‐mediated inflammatory responses, suggesting a feedback mechanism in immune regulation [9]. Furthermore, CRISPLD2 exhibits anti‐inflammatory effects in diverse inflammation‐related pathologies, including hepatic fibrosis [10], asthma [9], Gaucher disease [11], and obesity [7]. However, its specific role and the underlying mechanisms through which CRISPLD2 regulates disc inflammation in the context of IVDD remain unexplored.

Ferroptosis is a newly recognized form of regulated cell death driven by iron accumulation and the peroxidation of membrane lipids, particularly lipid hydroperoxides, leading to disruption of membrane integrity and cellular demise [12, 13]. Recent evidence had highlighted the involvement of ferroptosis in the development of IVDD [14]. Oxidative stress and elevated homocysteine levels, both of which are associated with disc degeneration, have been shown to suppress glutathione peroxidase 4 (GPX4), a key antioxidant enzyme that inhibits ferroptosis in NP cells, thereby promoting ferroptotic cell death [15, 16]. It is noteworthy that oxidative stress is closely related to ferroptosis because of a similar pathological process, and the crosstalk between oxidative stress and ferroptosis has been studied in some diseases, including ischemic stroke [17] and Alzheimer's disease [18]. Our previous work demonstrated that the interaction between ubiquitin specific protease 11 (USP11) and SIRT3 contributes to IVDD by regulating oxidative stress–induced ferroptosis [19]. A recent study revealed that CRISPLD2 was highly expressed in monocytes and may be involved in the regulation of ferroptosis [20]. However, whether CRISPLD2 modulates oxidative stress and ferroptosis crosstalk during IVDD progression and the mechanistic basis for such regulation have not yet been elucidated.

In this study, we identified CRISPLD2 as a key regulator of NPC homeostasis, mitigating oxidative stress‐induced ferroptosis and preventing excessive cellular damage in IVDD. Mechanistic investigations revealed that CRISPLD2 exerts its protective effects by modulating ferroptosis‐associated biomarkers and inflammatory pathways. Notably, we identified a CRISPLD2‐associated miR‐548I‐IL17A axis as a crucial mediator in this process, highlighting the interplay between inflammation and ferroptosis in IVDD pathogenesis. Our findings provide novel insights into the molecular mechanisms of IVDD and suggest that targeting the CRISPLD2‐miR‐548I‐IL17A axis may represent a promising therapeutic strategy for preventing disc degeneration and alleviating discogenic pain.

## 2 Results

### 2.1 Single‐Cell Transcriptome Profiling Reveals the Association of CRISPLD2 With IVDD and Ferroptosis

To uncover the cellular composition of NP tissue and identify cell type–specific gene expression changes associated with IVDD, we analyzed single‐cell RNA sequencing (scRNA‐seq) data from dataset GSE153066, which includes NP tissues obtained from 16 individuals, comprising 8 degenerative and 8 non‐degenerative samples. Uniform manifold approximation and projection (UMAP) visualization of all sequenced cells revealed distinct clusters representing diverse cell types within the NP microenvironment, including multiple NPC subpopulations, immune cells such as macrophages and neutrophils, and structural cell types such as endothelial cells and osteoclasts (Figure S1A). This cellular diversity underscored the complexity of the disc microenvironment and suggested that individual NPC subpopulations may perform specialized functions. Comparative analysis of healthy (CTL) and degenerated (IVDD) samples showed a marked shift in immune cell populations, with increased macrophages and neutrophils in IVDD tissues, supporting a central role for inflammation in disc degeneration (Figure S1B).

Differential gene expression analysis revealed that NPC1 exhibited the high COL2A1 and low COL1A1/MMP3 levels, indicating this subtype retained non‐degenerative features. In contrast, NPC3 and NPC4 displayed the elevated MMP13 and COL1A1 levels, along with the reduced COL2A1 expression, suggesting a degenerative phenotype (Figure S1C). Notably, compared with NPC3, the NPC4 subcluster exhibited even lower COL2A1 and higher COL1A1 expression, suggesting that NPC3 represents a mildly degenerative subtype, whereas NPC4 corresponds to a more advanced degenerative state. To further characterize NPC phenotypic transitions, we performed clustering analysis of NPC subtypes based on transcriptomic signatures, visualized by t‐distributed stochastic neighbor embedding (t‐SNE) (Figure S2A). Pseudotime trajectory analysis revealed a dynamic progression from progenitor‐like NPCs (NPC1) toward more differentiated and dysfunctional states (NPC3 and NPC4) during IVDD (Figure S2A,B). Proportional differences in NPC subtypes were observed, with NPC1 and NPC2 decreased in NP tissues, while NPC3, NPC4, NPC5, and NPC6 were increased, indicating subtype‐specific degeneration patterns (Figure S2C). Figure S2D presented the top five most highly and lowly expressed genes within each NPC subtype. Notably, pseudotime analysis of CRISPLD2 expression across subclusters revealed a dynamic change, in which CRISPLD2 was minimally expressed in NPC1, reached its peak level in NPC3 (mild degeneration), and then decreased again in NPC4 (advanced degeneration), indicating a stage‐dependent regulatory pattern during IVDD progression (Figure S2E). These findings suggested that CRISPLD2 may play a key role in the progression of disc degeneration (Figure S2F).

Gene Ontology (GO) enrichment analysis showed iron‐related terms, including “response to iron ion,” “iron transport,” and “mitochondrial iron homeostasis,” were enriched in NPC3 and NPC4, implicating iron dysregulation in IVDD (Figure S3A). Similarly, lipid metabolism‐related GO terms were also enriched in these subtypes, suggesting lipid metabolic reprogramming is implicated in disease progression (Figure S3B). Given prior evidence linking iron and lipid metabolism to ferroptosis, we evaluated ferroptosis pathway activity (WP_FERROPTOSIS) across NPC subtypes. The Kruskal‐Wallis test revealed significant differences in pathway activation among subtypes (*p* < 2.2e‐16) (Figure S3C), supporting a subtype‐specific ferroptotic vulnerability. Correlation matrix analysis identified associations between CRISPLD2 and key ferroptosis‐related genes, including acyl‐CoA synthetase long‐chain family member 4 (ACSL4), glutathione peroxidase 4 (GPX4), ferritin heavy chain 1 (FTH1), heme oxygenase 1 (HMOX1), NADPH oxidase 4 (NOX4), highlighting its potential role in regulating oxidative stress and ferroptotic cell death in IVDD (Figure S3D). Collectively, these findings suggested that CRISPLD2 is a potential regulator of ferroptosis in degenerative NPCs, contributing to the pathogenesis of IVDD.

### 2.2 CRISPLD2 Expression is Downregulated in Severely Degenerative NPCs

**FIGURE 1:**
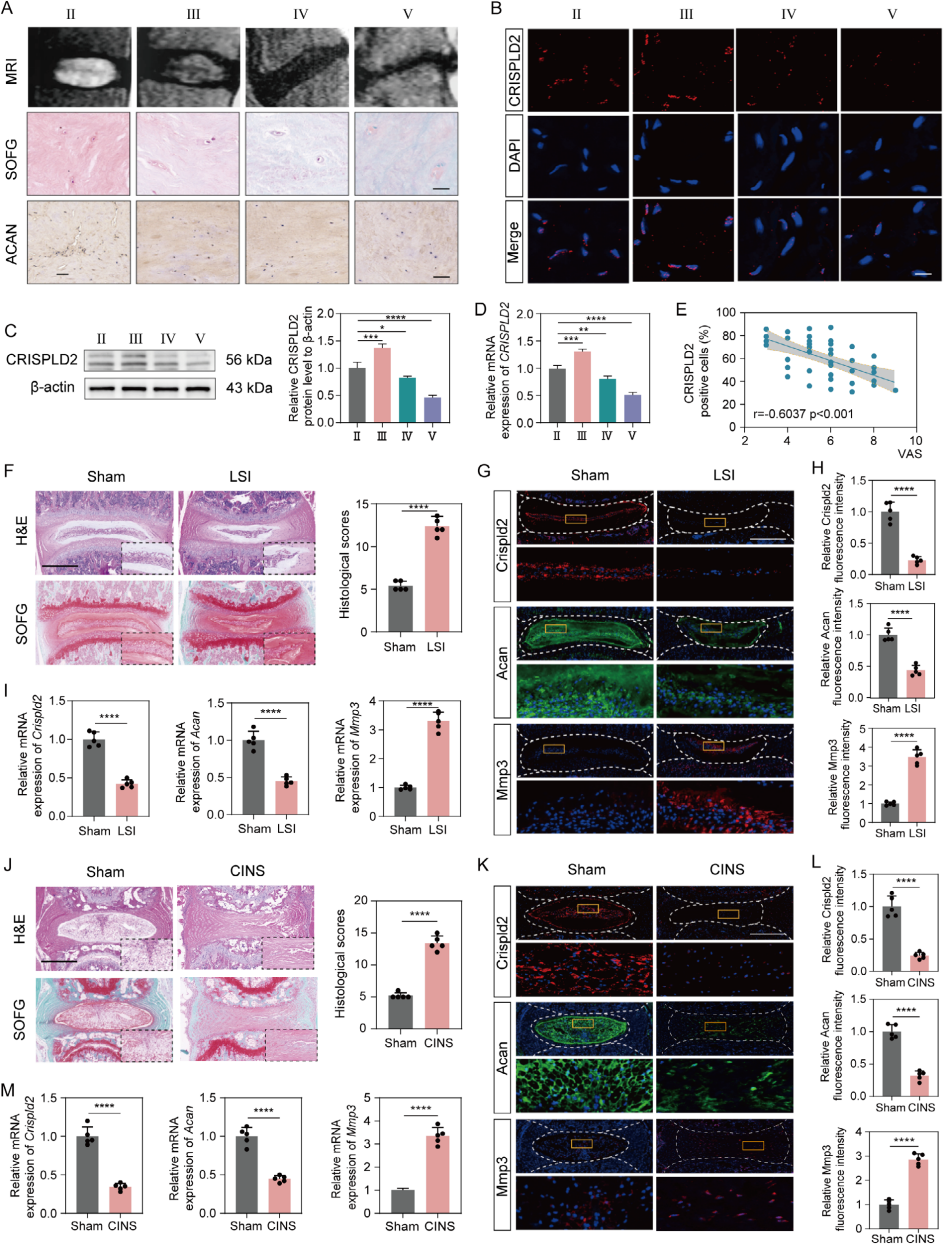
CRISPLD2 expression was decreased in severely degenerative NPCs and correlates with patient pain severity. (A) Representative MRI, SOFG, and ACAN IHC staining of NP tissues at Pfirrmann grades II–V. Progressive loss of disc height and matrix components was observed with increasing degeneration. Scale bars: 100 µm (SOFG), 200 µm (ACAN). (B) IF staining of CRISPLD2 in NP samples showing decreased CRISPLD2‐positive cells in severely degenerated tissues. Scale bar: 100 µm. (C) Western blot analysis of CRISPLD2 protein level in NP tissues across degeneration stages and corresponding quantification. (D) The RT‐qPCR analysis showing relative mRNA expression of *CRISPLD2* in grades II–V. (E) Pearson correlation between CRISPLD2 expression and patient‐reported VAS pain scores. (F) H&E and SOFG staining of disc sections from sham and LSI mouse. Scale bar: 500 µm. (G) IF staining of Crispld2, Acan, and Mmp3 in sham and LSI model. Scale bar: 500 µm. (H) Quantification of fluorescence intensity of Crispld2, Acan, and Mmp3. (I) RT‐qPCR analyses of *Crispld2*, *Acan*, and *Mmp3* expression in NP tissues from sham and LSI model. (J) H&E and SOFG staining of disc sections from sham and CINS mouse. Scale bar: 500 µm. (K) IF staining of Crispld2, Acan, and Mmp3 in sham and CINS model. Scale bar: 500 µm. (L) Quantification of fluorescence intensity of Crispld2, Acan, and Mmp3. (M) RT‐qPCR analyses of *Crispld2*, *Acan*, and *Mmp3* expression in NP tissues from sham and CINS model. Data are presented as mean ± standard deviation (SD), *n* = 5. Statistical analysis was performed using one‐way ANOVA followed by Tukey's post hoc test. *p < 0.05, **p < 0.01, ***p < 0.001, ****p < 0.0001.

To clarify the role of CRISPLD2 in the pathogenesis of IVDD, we assessed its expression in human NP tissues collected from patients undergoing discectomy. The degree of degeneration was classified using the Pfirrmann grading system based on preoperative T2‐weighted MRI scans (**Figure** **1**A). Histological staining, including Safranin O & Fast Green (SOFG), and immunohistochemistry (IHC) for ACAN, was employed to assess matrix composition, demonstrating a significant reduction in proteoglycan content in more degenerated samples (Figure 1A, Figure S4A). Immunofluorescence (IF) staining further revealed a progressive decrease in CRISPLD2‐positive cells, particularly in advanced degeneration stages (Figure 1B, Figure S4B). Western blot (WB) revealed that the levels of CRISPLD2 were slightly elevated in the mildly degenerated NP samples (Pfirrmann grade III), whereas showed a marked decrease for severely degenerated tissues (Pfirrmann grade IV and V) (Figure 1C). The RT‐qPCR results showed the same trends (Figure 1D). Notably, a negative correlation was observed between CRISPLD2 levels and patient‐reported Visual Analog Scale (VAS) scores (Figure 1E), suggesting that CRISPLD2 expression may influence pain perception in degenerative conditions.

To determine whether Crispld2 expression varies in IVDD mouse models of different severities, we established two commonly used mouse models of disc degeneration: the lumbar spine instability (LSI) model and the caudal intervertebral needle‐stab (CINS) model. Histological evaluation using Hematoxylin and Eosin (H&E) and SOFG staining revealed disorganization of the AF‐NP boundary and a marked reduction in NP cell number in both models, confirming successful IVDD model induction (Figure 1F,J). IF staining revealed a significant downregulation of Acan and upregulation of Mmp3 in both LSI and CINS groups (Figure 1G,H,K,L). Importantly, consistent with findings from human degenerative disc samples, Crispld2 expression was significantly downregulated in the NP tissues of both models, as shown by IF staining (Figure 1G,H,K,L). The RT‐qPCR analysis of the mRNA expression of *Acan*, *Mmp3* and *Crispld2* was consistent with the IF results (Figure 1I,M). Taken together, these findings demonstrated that CRISPLD2 expression is closely associated with the severity of IVDD, providing a foundation for further mechanistic studies on its regulatory role in disc pathology.

### 2.3 High Concentration or Prolonged IL‐1β Exposure Reduced CRISPLD2 Expression in NPCs

IL‐1β is a key proinflammatory cytokine implicated in the pathogenesis of IVDD and is commonly used to induce disc degeneration in vitro [21]. To further examine the regulatory pattern of CRISPLD2 under inflammatory conditions, we treated human NPCs with IL‐1β at varying concentrations and durations, establishing an in vitro model of disc degeneration. In the time‐course experiment, IL‐1β (10 ng/mL) exposure resulted in a significant increase in the expression protein level of CRISPLD2 at 24 h, whereas prolonged exposure time resulted in a significant decrease in the CRISPLD2 level (Figure S5A,B). Furthermore, western blot analysis revealed a time‐dependent decrease in ACAN and COL2A1 expression, accompanied by a marked increase in MMP3 and ADAMTS5 levels (Figure S5A,B). These findings were further supported by RT‐qPCR and IF staining results (Figure S5C,D).

In the dose‐course experiment, the protein levels of CRISPLD2 were remarkably raised by 10 ng/mL IL‐1β, which were significantly decreased by higher concentrations of IL‐1β (Figure S6A,B). A dose‐dependent decrease in ACAN and COL2A1 protein levels, accompanied by an increase in MMP3 and ADAMTS5 expression, was also observed (Figure S6A,B). RT‐qPCR analysis and IF staining further verified the results of WB (Figure S6C,D). These results collectively suggested that IL‐1β significantly modulates key markers associated with ECM remodeling in a time‐ and dose‐dependent manner, and that prolonged exposure to IL‐1β or high level of IL‐1β could remarkably decrease the expression level of CRISPLD2.

### 2.4 Deficiency of Crispld2 Promotes IVDD and Poor Pain‐Related Behavioral Scores

**FIGURE 2:**
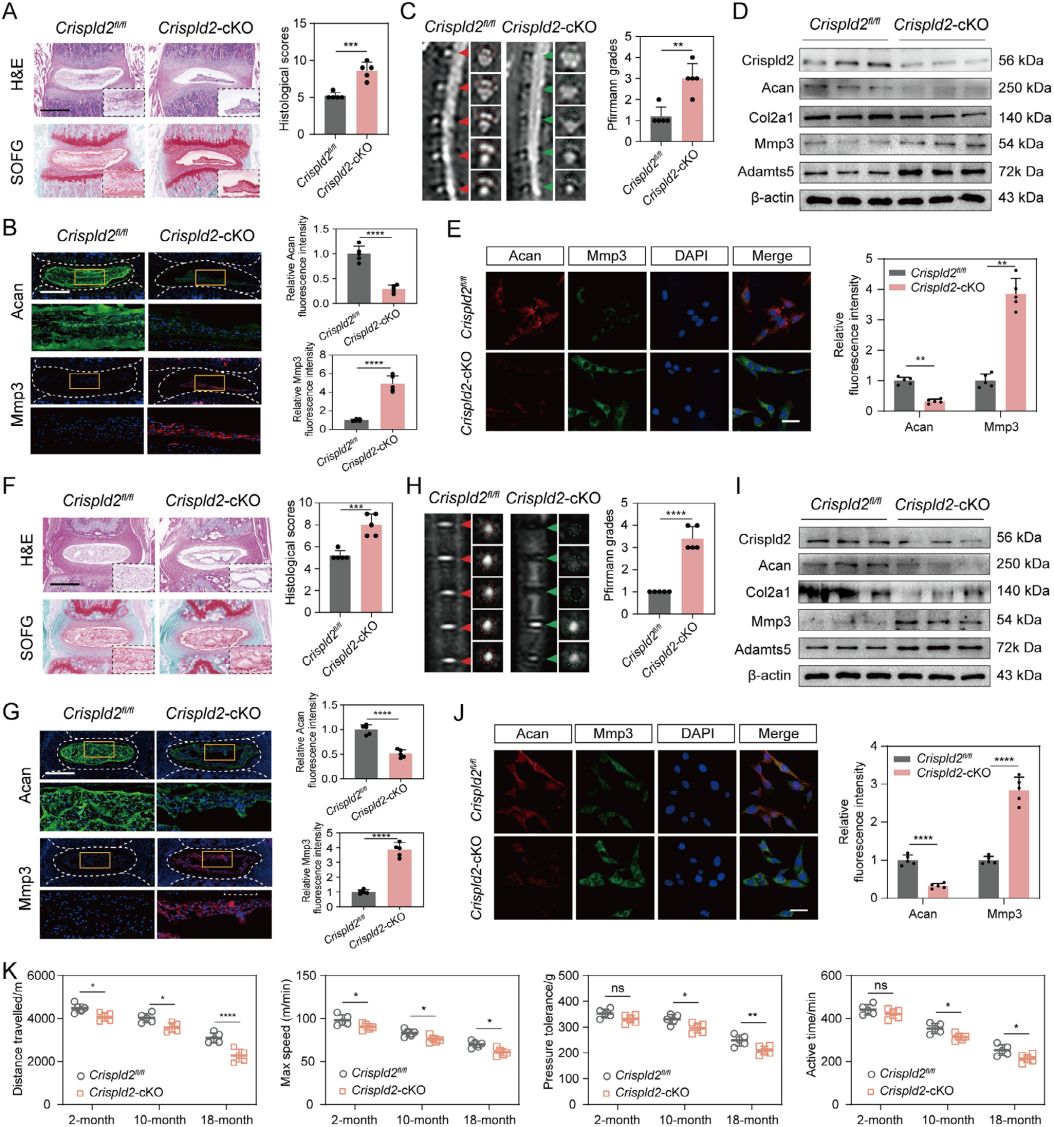
Crispld2 deficiency exacerbates IVDD and associated pain‐like behaviors. (A) Representative H&E and SOFG staining of lumbar discs from the *Crispld2^fl/fl^* and *Crispld2*‐cKO mice. Histological scores were significantly elevated in *Crispld2*‐cKO discs. Scale bar: 500 µm. (B) IF staining of Acan and Mmp3 in lumbar disc sections. Quantification of fluorescence intensity indicates decreased Acan and increased Mmp3 in *Crispld2*‐cKO discs. Scale bar: 500 µm. (C) Representative MRI images and corresponding Pfirrmann grading show reduced T2‐weighted signal intensity and increased degeneration scores in *Crispld2*‐cKO discs lumbar discs. (D) Western blot analysis of lumbar disc tissue. (E) IF staining of primary NPCs from the *Crispld2^fl/fl^* and *Crispld2*‐cKO discs mice. Scale bar: 50 µm. (F) H&E and SOFG staining of coccygeal discs in the *Crispld2^fl/fl^* and *Crispld2*‐cKO mice. Scale bar: 500 µm. (G) IF of coccygeal discs. Scale bar: 500 µm. (H) MRI imaging and Pfirrmann scores of coccygeal discs. (I) Western blot analysis of coccygeal discs recapitulates lumbar findings. (J) IF of primary NPCs from coccygeal discs. Scale bar: 50 µm. (K) Behavioral analysis across 2‐, 10‐, and 18‐month‐old mice. Data are presented as mean ± SD (*n* = 5). Statistical comparisons were performed using unpaired two‐tailed Student's *t*‐test or one‐way ANOVA with Tukey's post hoc test. *p < 0.05, **p < 0.01, ***p < 0.001, ****p < 0.0001.

To investigate whether *Crispld2* deficiency in intervertebral discs contributes to the progression of IVDD, NP‐specific *Crispld2*‐cKO mice were established. Both lumbar and coccygeal discs were examined in parallel to ensure consistent observations across spinal levels. Histological analysis of both lumbar and caudal intervertebral discs using H&E and SOFG staining revealed pronounced matrix disorganization and proteoglycan loss in the *Crispld2*‐cKO group, with significantly higher histological scores compared to *Crispld2^fl/fl^* controls (**Figure** **2**A,F). IF staining further showed that *Crispld2* deficiency led to a marked reduction of Acan and a concomitant increase in Mmp3 expression in the NP region of both lumbar and coccygeal discs (Figure 2B,G). Consistent with the histological changes, MRI imaging demonstrated reduced signal intensity in both discs of *Crispld2*‐cKO mice, reflecting disc dehydration and structural compromise after *Crispld2* deletion (Figure 2C,H). The Pfirrmann grading score further confirmed the above result (Figure 2C,H). The WB results of NP tissues from lumbar and coccygeal discs revealed markedly reduced levels of Crispld2, Acan, and Col2a1, along with elevated expression of Mmp3 and Adamts5 in *Crispld2*‐cKO mice, confirming molecular features of matrix degradation (Figure 2D,I). Quantitative analysis of the protein levels was presented in Figure S7. The co‐immunofluorescence of Acan and Mmp3 for the primary NPCs from the *Crispld2^fl/fl^* and *Crispld2*‐cKO mice further confirmed the above findings (Figure 2E,J). Additionally, behavioral assessments of the *Crispld2^fl/fl^* and *Crispld2*‐cKO mice at 2, 10, and 18 months revealed a significant decline in functional mobility (both distance travel and max speed), pressure tolerance, and active time in the *Crispld2*‐cKO group, indicating *Crispld2* deficiency‐induced disc degeneration might result in hyperalgesia (Figure 2K). Collectively, these results demonstrated that Crispld2 is essential for maintaining intervertebral disc structural integrity and ECM homeostasis, and that its deficiency leads to progressive disc degeneration and associated pain‐like behaviors.

### 2.5 The Deficiency of Crispld2 Promotes Mitochondrial Dysfunction and Oxidative Stress–Induced Ferroptosis Within NPCs

**FIGURE 3:**
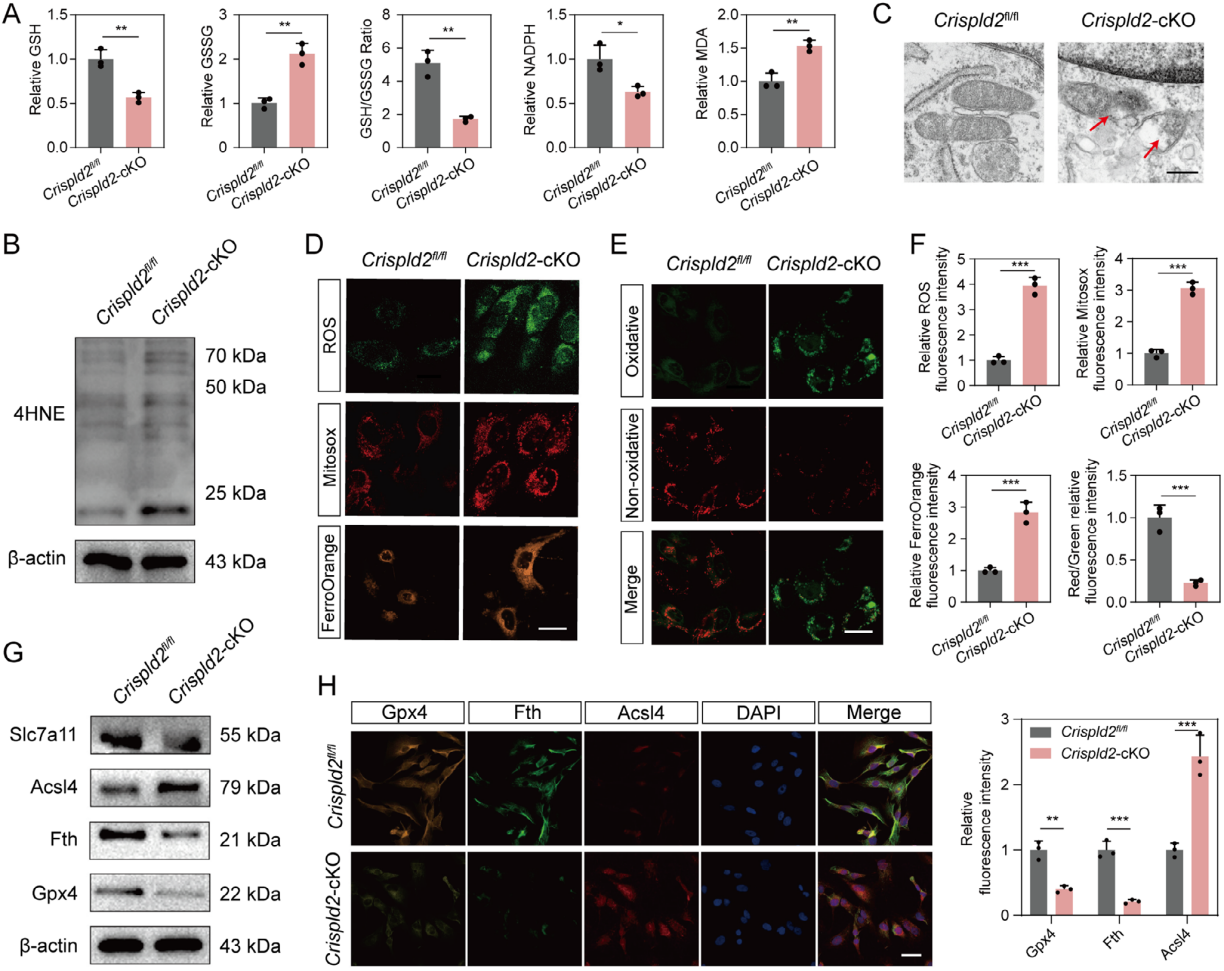
Crispld2 deficiency promotes ferroptosis and mitochondrial oxidative stress in NPCs. (A) Quantification of oxidative stress indicators in *Crispld2^fl/fl^* and *Crispld2*‐cKO NPCs, including GSH, GSSG, GSH/GSSG, NADPH, and MDA. (B) Western blot analysis of 4HEN *Crispld2^fl/fl^* and *Crispld2*‐cKO NPCs. (C) TEM of mitochondria in NPCs from *Crispld2^fl/fl^* and *Crispld2*‐cKO mice. Red arrows indicate damaged mitochondria. Scale bar: 50 nm. (D) Representative fluorescence images of intracellular ROS, mitochondrial ROS (MitoSOX), and FerroOrange in *Crispld2^fl/fl^* and *Crispld2*‐cKO NPCs. Scale bar: 20 µm. (E) Representative fluorescence images of Lipid peroxidation in *Crispld*2*^fl/fl^* and *Crispld2*‐cKO NPCs. Scale bar: 20 µm. (F) Quantification of fluorescence intensity. (G) Western blot analysis of ferroptosis‐related markers in *Crispld2^fl/fl^* and *Crispld2*‐cKO NPCs. (H) Representative immunofluorescence images and quantification of Gpx4, Acsl4, and Fth. Scale bar: 50 µm. Data are presented as mean ± SD (*n* = 3). Statistical comparisons were performed using two‐tailed unpaired Student's *t*‐test. *p < 0.05, **p < 0.01, ***p < 0.001, ****p < 0.0001, ns = not significant.

Oxidative stress, triggered by an imbalance between reactive oxygen species (ROS) production and the antioxidant defense system, plays a key role in ferroptosis [22, 23]. To explore whether Crispld2 is involved in regulating oxidative stress, we first assessed the mitochondrial function and redox homeostasis in NPCs from *Crispld2*‐cKO mice. Compared with controls, *Crispld2*‐deficient NPCs exhibited markedly reduced levels of glutathione (GSH) and nicotinamide adenine dinucleotide phosphate (NADPH), alongside an increase in oxidized GSH (GSSG), reflecting depletion of antioxidant capacity and redox imbalance (**Figure** **3**A). Moreover, the level of the oxidative stress marker malondialdehyde (MDA) was significantly elevated in *Crispld2*‐cKO group, suggesting enhanced oxidative damage (Figure 3A). Consistently, the WB result revealed that the expression of 4‐hydroxynonenal (4HNE), a well‐recognized marker of oxidative stress‐induced lipid peroxidation, was also increased following *Crispld2* deletion (Figure 3B). Transmission electron microscopy (TEM) further revealed the mitochondrial morphological abnormalities in *Crispld2*‐deficient NPCs, including shrinkage, cristae loss, and increased membrane density, underscoring impaired mitochondrial function (Figure 3C).

To determine whether oxidative stress induced by Crispld2 loss leads to ferroptosis, we further evaluated the reactive oxygen species (ROS) levels and ferroptosis‐related markers. Fluorescence staining showed that levels of intracellular ROS, mitochondrial ROS (MitoSOX), and ferroptosis markers (FerroOrange) were significantly increased after *Crispld2* cKO, suggesting that ferroptosis was activated in the absence of *Crispld2* (Figure 3D,F). The BODIPY staining also indicated elevated lipid peroxidation in *Crispld2*‐deficient cells (Figure 3E,F). WB results demonstrated decreased expression of Slc7a11, Gpx4 and Fth (key negative regulators of ferroptosis), and increased levels of Acsl4 (a pro‐ferroptotic enzyme) in *Crispld2*‐cKO group (Figure 3G, Figure S8A–D). These findings were further confirmed by IF staining (Figure 3H, Figure S8E,F). Collectively, these findings indicated that Crispld2 deficiency disrupts mitochondrial integrity and antioxidant homeostasis, leading to oxidative stress‐driven ferroptosis in NP cells.

### 2.6 IL17A Pathway is Activated Following CRISPLD2 Downregulation

**FIGURE 4:**
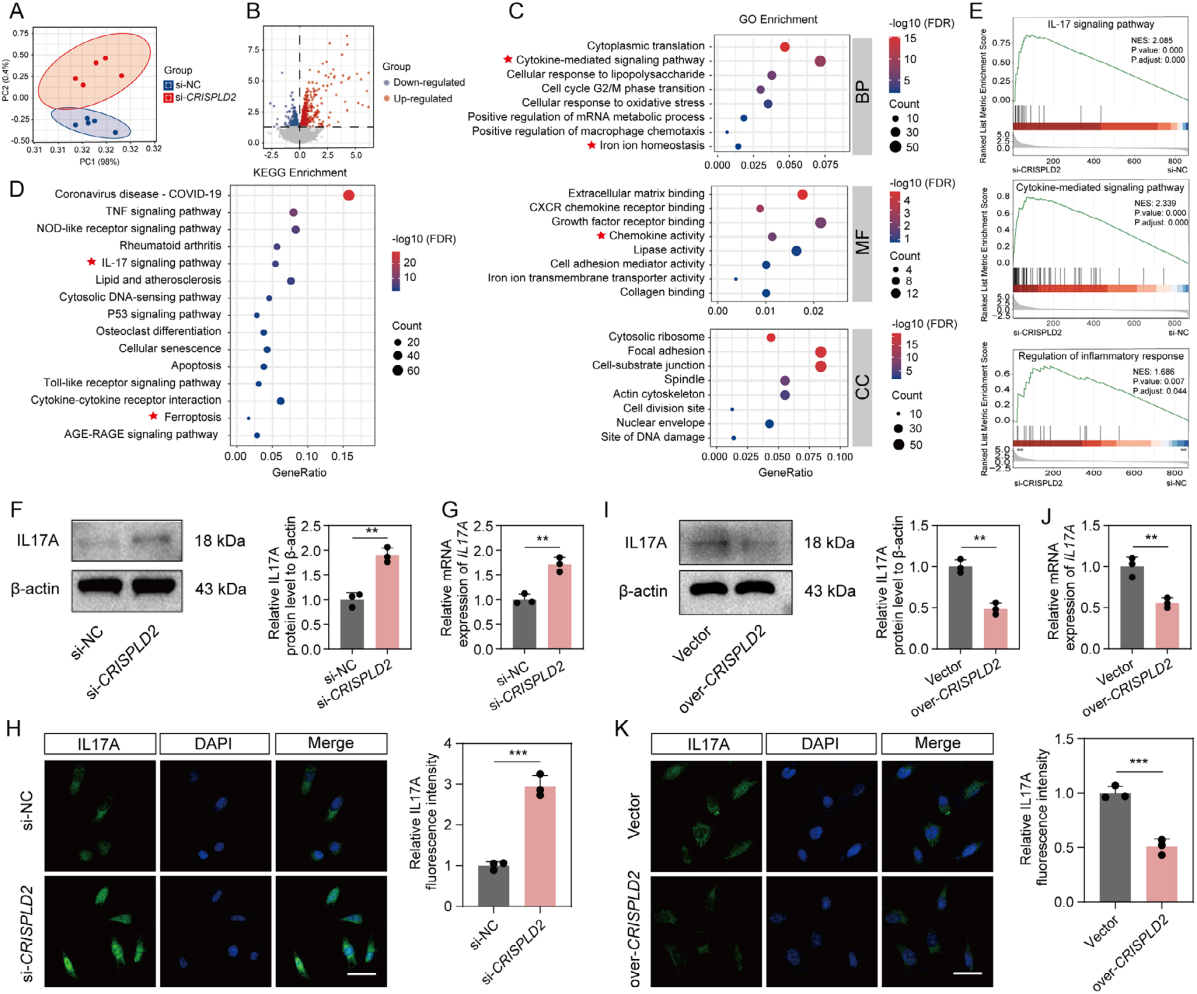
Downregulation of CRISPLD2 activates IL17A signaling pathway in NPCs. (A) PCA showing distinct clustering of transcriptomic profiles between si‐NC and si‐*CRISPLD2* groups. (B) Volcano plot illustrating DEGs between groups. (C) GO enrichment analysis of DEGs. (D) KEGG pathway enrichment of DEGs. (E) GSEA confirming IL17 signaling, cytokine‐mediated signaling, and inflammatory response regulation. (F,G) Western blot and RT‐qPCR analysis showing increased IL17A protein and mRNA levels in si‐*CRISPLD2* versus si‐NC groups. (H) Immunofluorescence staining and quantification of IL17A. Scale bar: 50 µm. (I,J) Western blot and RT‐qPCR analyses showing decreased IL17A expression in *CRISPLD2*‐overexpressing cells. (K) Immunofluorescence staining showing decreased IL17A fluorescence intensity in over‐*CRISPLD2* group. Scale bar: 50 µm. Data are presented as mean ± SD (*n* = 3). Statistical comparisons were performed using unpaired two‐tailed Student's *t*‐test. *p < 0.05, **p < 0.01, ***p < 0.001, ****p < 0.0001.

To elucidate the molecular mechanism by which CRISPLD2 modulates NPCs function, we performed high‐throughput RNA sequencing (RNA‐seq) in human NPCs transfected with si‐*CRISPLD2* or control siRNA (si‐NC). Principal component analysis (PCA) revealed distinct clustering between the two groups, indicating substantial transcriptomic alterations following *CRISPLD2* knockdown (**Figure** **4**A). A total of 873 differentially expressed genes (DEGs) were identified, with 500 upregulated and 373 downregulated transcripts, as illustrated in the volcano plot (Figure 4B). Gene Ontology (GO) enrichment analysis showed that the DEGs were significantly enriched in biological processes (BP) related to cytokine‐mediated signaling, cellular response to lipopolysaccharide, and iron ion homeostasis (Figure 4C). In terms of molecular function (MF), DEGs were notably enriched in chemokine activity and CXCR chemokine receptor binding (Figure 4C). For cellular components (CC), enrichment was observed in ECM‐related structures such as cell‐substrate junctions and mitotic spindle components (Figure 4C). These results suggested that CRISPLD2 may be involved in regulating ECM metabolism, inflammation, and oxidative stress in the context of IVDD. Furthermore, KEGG pathway enrichment analysis revealed an upregulation of genes enriched in the IL17 signaling pathway and ferroptosis following *CRISPLD2* silencing (Figure 4D). Gene Set Enrichment Analysis (GSEA) further confirmed upregulation of pathways associated with IL17 signaling, cytokine activity, and inflammatory response in the *CRISPLD2* knockdown group (Figure 4E).

Our results of KEGG and GSEA analyses revealed that the IL17A signaling pathway was among the prominently upregulated pathways following CRISPLD2 depletion. We prioritized this pathway for mechanistic investigation due to IL17A's well‐established, potent pro‐inflammatory role in IVDD pathogenesis and prior reports linking CRISPLD2 to other interleukins [24, 25, 26]. Since IL17A has also been reported to influence ferroptosis and oxidative stress in other diseases [27, 28], it was speculated that CRISPLD2 may regulate oxidative stress‐induced ferroptosis in IVDD through IL17A signaling. To test this, we investigated the regulatory effect of CRISPLD2 on IL17A expression in human NPCs. Western blot and RT‐qPCR analyses showed that CRISPLD2 knockdown significantly increased IL17A mRNA and protein levels (Figure 4F,G), which was further supported by the enhanced immunofluorescence staining in CRISPLD2‐silenced NPCs (Figure 4H). In contrast, overexpression of CRISPLD2 resulted in a marked reduction in IL17A expression at both the mRNA and protein levels (Figure 4I,J), accompanied by the decreased immunofluorescence signal intensity (Figure 4K). Collectively, these findings suggested that CRISPLD2 negatively regulates IL‐17A expression.

### 2.7 CRISPLD2 Regulates Ferroptosis by Modulating the Expression of IL17A

**FIGURE 5:**
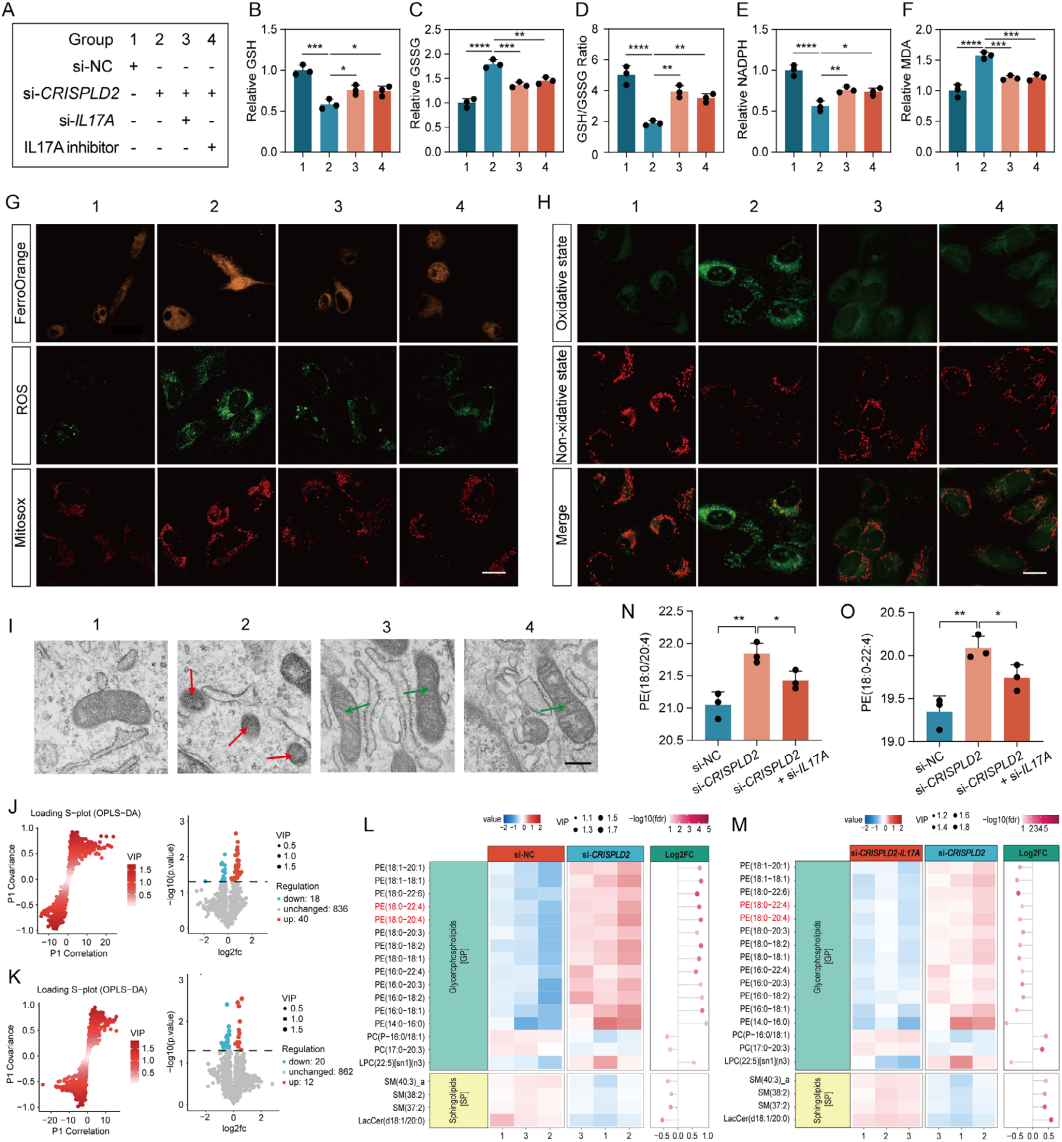
CRISPLD2 inhibits ferroptosis in NPCs by suppressing IL‐17A–mediated lipid peroxidation and oxidative stress. (A) Schematic representation of experimental groups: (1) si‐NC; (2) si‐*CRISPLD2*; (3) si‐*CRISPLD2* + si‐*IL17A*; and (4) si‐*CRISPLD2* + IL‐17A inhibitor. (B–F) Biochemical assessment of ferroptosis‐associated redox balance. (G) Representative fluorescence images of FerroOrange, ROS, and MitoSOX staining. Scale bar: 20 µm. (H) Immunofluorescence analysis of lipid peroxidation states. Scale bar: 20 µm. (I) TEM of mitochondria. Red arrows indicate damaged mitochondria, characterized by darker matrix and loss of cristae. Green arrows indicate partially restored mitochondrial cristae. Scale bar: 50 nm. (J,K) Analysis of loading S‐plot (OPLS‐DA). (L,M) Heatmaps of oxidized phosphatidylethanolamine (PE) species and other lipid classes. (N,O) Quantification of PE (18:0/20:4) and PE (18:0/22:4) levels. Data represent mean ± SD (*n* = 3). Statistical comparisons were performed using one‐way ANOVA with Tukey's post hoc test. *p < 0.05, **p < 0.01, ***p < 0.001, ****p < 0.0001.

According to the above findings, it was speculated that IL17A and ferroptosis might play pivotal roles in CRISPLD2 deficiency associated IVDD. However, the specific mechanism was not clear. Thus, the following experiments were carried out to explore the potential mechanisms. The experimental groups used in the study were outlined, which included group 1 (si‐NC), group 2 (si‐*CRISPLD2*), group 3 (si‐*CRISPLD2*+si‐*IL17A*), and group 4 (si‐*CRISPLD2*+IL17A inhibitor) (**Figure** **5**A). We analyzed the balance between oxidative stress and cellular antioxidant capacity by quantifying GSH levels, GSH/GSSG ratios, and the expression of key metabolic enzymes, including MDA and NADPH. The results showed significant reductions in GSH and NADPH levels and increases in GSSG and MDA in si‐*CRISPLD2* group, indicating that *CRISPLD2* downregulation triggered oxidative stress, whereas *IL17A* downregulation or inhibition remarkably promoted shifts toward a less oxidized state (Figure 5B–F). FerroOrange staining further confirmed a significant increase in iron accumulation in the si‐*CRISPLD2* group, which was notably reduced upon *IL17A* silencing or inhibition (Figure 5G, Figure S9A). Similarly, ROS and MitoSOX levels were significantly elevated in si‐*CRISPLD2* cells but attenuated in both IL17A‐targeted groups (Figure 5G, S9B–C). BODIPY assay analysis revealed increased oxidative lipid damage in the si‐*CRISPLD2* group, whereas *IL17A* knockdown or inhibition led to a clear shift toward a reduced oxidative state (Figure 5H, S9D). The TEM results showed mitochondrial swelling and a significant reduction in cristae in NPCs of the si‐*CRISPLD2* group, which were largely ameliorated by IL17A suppression (Figure 5I). These findings demonstrated that CRISPLD2 regulates ferroptosis by modulating IL17A expression, and that targeting IL17A can effectively alleviate the detrimental effects of CRISPLD2 deficiency during IVDD.

Given that the accumulation of lipid peroxides derived from polyunsaturated fatty acids (PUFAs) is the central biochemical event that defines ferroptosis, we focused our investigation on the lipid metabolism. This approach allowed us to directly test whether CRISPLD2 modulates the core execution pathway of ferroptosis before exploring other upstream regulatory factors. To further explore the involvement of lipid metabolic alterations in CRISPLD2‐mediated ferroptosis, we performed comprehensive lipidomic analyses under two conditions: (1) *CRISPLD2* knockdown versus negative control (si‐*CRISPLD2* vs. si‐NC), and (2) combined knockdown of *CRISPLD2* and *IL17A* versus *CRISPLD2* knockdown alone (si‐*CRISPLD2*‐*IL17A* vs. si‐*CRISPLD2*) in human NPCs. Volcano plot analysis identified 40 upregulated and 18 downregulated lipid species following *CRISPLD2* knockdown compared to the control group (Figure 5J). In contrast, co‐silencing of *CRISPLD2* and *IL17A* led to 12 upregulated and 20 downregulated lipid species relative to *CRISPLD2* knockdown alone (Figure 5K). A total of 20 overlapping differentially expressed lipids were shared between the two comparisons and selected for further investigation (Figure 5L,M). Among these, phosphatidylethanolamines PE (18:0/20:4) and PE (18:0/22:4), previously identified as highly susceptible to lipid peroxidation and strongly implicated in ferroptosis [29], were significantly elevated in response to *CRISPLD2* silencing. Notably, this elevation was reversed upon simultaneous *IL17A* knockdown (Figure 5L–O). Collectively, our findings established that CRISPLD2 deficiency promoted ferroptosis by enhancing IL17A‐driven phospholipid peroxidation and impairing mitochondrial oxidative homeostasis in disc cells.

### 2.8 CRISPLD2 Modulates IL17A via miR‐548I‐Mediated Targeting

**FIGURE 6:**
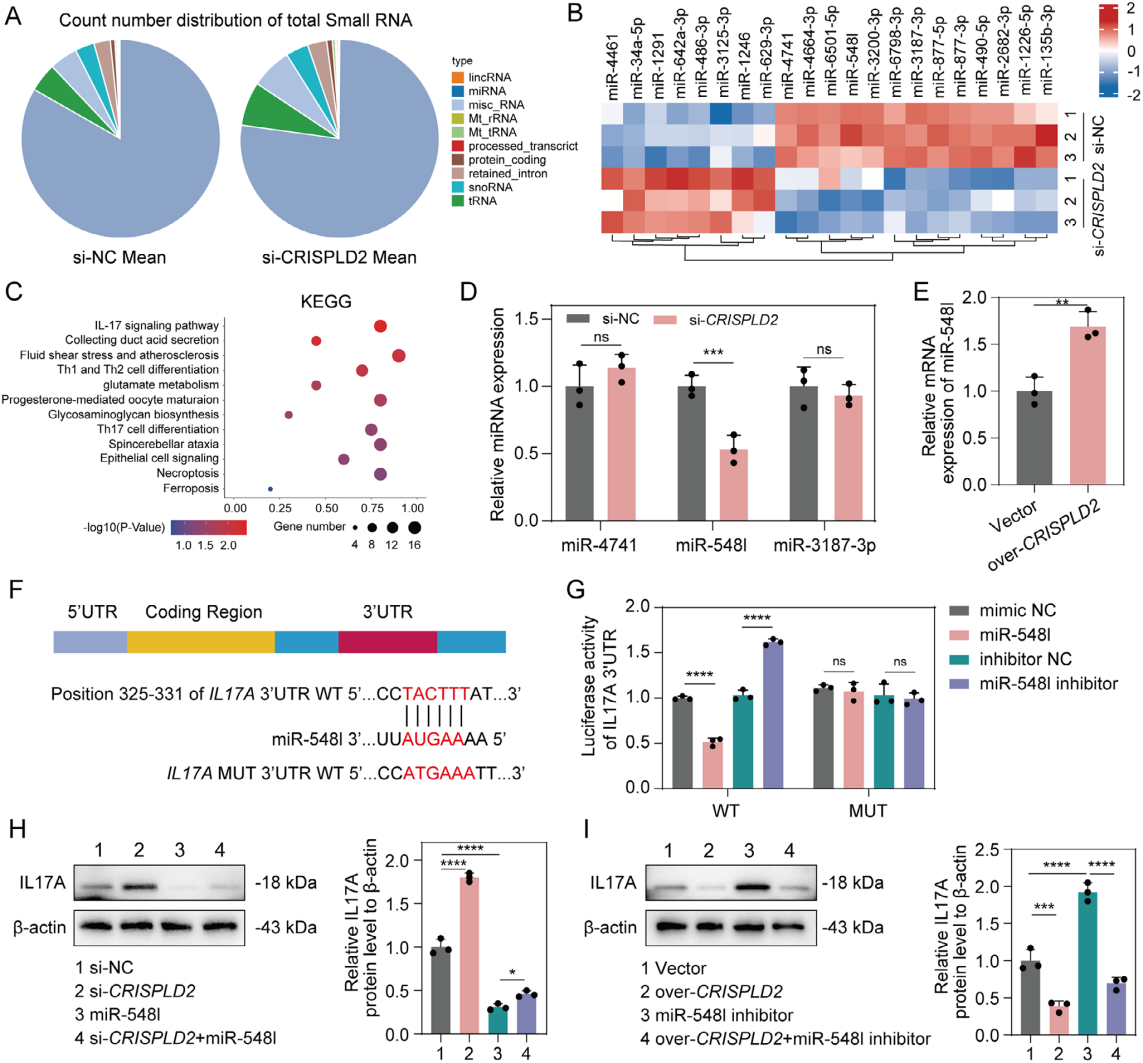
CRISPLD2 regulates IL‐17A expression through miR‐548I‐mediated post‐transcriptional suppression. (A) Pie charts showing the distribution of small RNA species in si‐NC and si‐*CRISPLD2* groups. (B) Heatmap illustrating differentially expressed miRNAs upon *CRISPLD2* knockdown. (C) KEGG pathway enrichment of differentially expressed miRNAs highlights the IL‐17 signaling pathway and ferroptosis as significantly impacted. (D) RT‐qPCR validation of selected miRNAs shows reduced miR‐548I expression following CRISPLD2 knockdown. (E) Overexpression of *CRISPLD2* significantly upregulates miR‐548I expression. (F) Predicted binding site of miR‐548I in the 3'‐UTR of *IL‐17A* mRNA, indicating direct interaction potential. (G) Dual‐luciferase reporter assay confirms that miR‐548I reduces *IL‐17A* 3'‐UTR luciferase activity in WT constructs, but not in MUT constructs. (H) Western blot and quantification of IL‐17A protein expression in NPCs co‐treated with si‐*CRISPLD2* and/or miR‐548I mimic. (I) Western blot showing that *CRISPLD2* overexpression suppresses IL‐17A, whereas miR‐548I inhibitor rescues IL‐17A expression. Data are presented as mean ± SD (*n* = 3). Statistical comparisons were performed using one‐way ANOVA followed by Tukey's post hoc test or unpaired two‐tailed Student's *t*‐test as appropriate. *p < 0.05, **p < 0.01, ***p < 0.001, ****p < 0.0001, ns = not significant.

MicroRNAs (miRNAs) play essential roles in various pathological conditions, including intervertebral disc degeneration (IVDD), and are increasingly recognized as promising therapeutic targets [30, 31]. To investigate whether miRNAs are involved in CRISPLD2‐mediated effects, we examined miRNA expression in human NPCs following *CRISPLD2* knockdown. The results showed a significant alteration in miRNA expression profiles in response to *CRISPLD2* silencing (**Figure** **6**A), with 8 miRNAs upregulated and 13 miRNAs downregulated in the si‐*CRISPLD2* group (Figure 6B). To explore the functional significance of these differentially expressed miRNAs, KEGG pathway enrichment analysis was performed, revealing several significantly affected pathways, most notably the IL17A signaling pathway, which was consistent with the mRNA‐seq result obtained from *CRISPLD2*‐silenced human NPCs (Figure 6C). These findings suggested that some of the altered miRNAs may be involved in the regulation of IL17A signaling. Given that miRNAs primarily function by promoting mRNA degradation [32], and considering that *IL17A* mRNA was upregulated upon *CRISPLD2* knockdown, we hypothesized that *CRISPLD2* deficiency may lead to the downregulation of miRNAs targeting *IL17A*. Using the TargetScan database (https://www.targetscan.org/vert_80/), we identified miR‐4741, miR‐548I, and miR‐3187‐3p as potential candidates targeting IL17A. We next measured the expression levels of these three miRNAs following *CRISPLD2* knockdown. Notably, miR‐548I was significantly downregulated in the si‐*CRISPLD2* group, whereas no significant changes were observed in miR‐4741 or miR‐3187‐3p (Figure 6D). To confirm whether CRISPLD2 directly modulates miR‐548I, we overexpressed *CRISPLD2* in NPCs, which led to a marked increase in miR‐548I expression (Figure 6E), further supporting a regulatory role of CRISPLD2 in controlling miR‐548I levels.

Database predictions indicated that miR‐548I has the potential to bind the 3'‐UTR of IL17A (Figure 6F). To validate this interaction, luciferase reporter constructs containing the wild‐type or mutated IL17A 3'‐UTR were generated and transfected into 293T cells. Luciferase activity was significantly reduced by the miR‐548I mimic and increased by the miR‐548I inhibitor. This effect was abolished when the IL17A 3'‐UTR was mutated (Figure 6G), confirming that miR‐548I directly binds to the 3'‐UTR of IL17A and represses its expression. To further clarify the role of miR‐548I in the regulatory relationship between CRISPLD2 and IL17A, we evaluated IL17A protein expression under various conditions. Overexpression of miR‐548I significantly attenuated the upregulation of IL17A induced by *CRISPLD2* knockdown (Figure 6H). In contrast, *CRISPLD2* overexpression suppressed IL17A protein levels, an effect that was substantially reversed by inhibition of miR‐548I (Figure 6I). Together, these findings suggested that CRISPLD2 suppresses IL17A expression by upregulating miR‐548I, establishing a CRISPLD2–miR‐548I–IL17A regulatory axis.

### 2.9 MiR‐548I Inhibits Ferroptosis by Regulating IL17A

**FIGURE 7:**
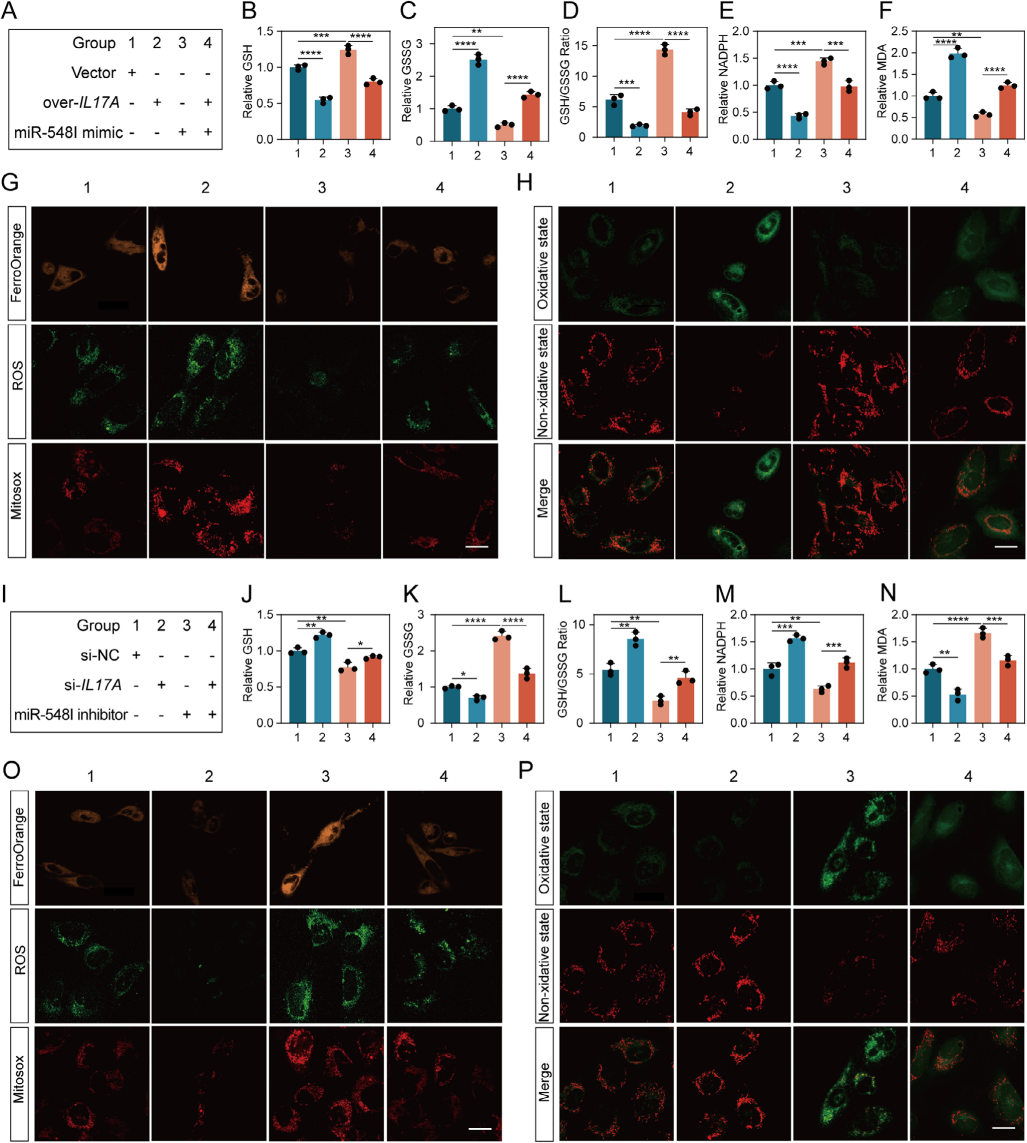
MiR‐548I inhibits ferroptosis in NPCs by suppressing IL‐17A‐mediated oxidative stress and mitochondrial dysfunction. (A) Experimental design for NPC treatment groups: (1) vector; (2) overexpression of *IL‐17A*; (3) miR‐548I mimic; (4) overexpression of *IL‐17A* + miR‐548I mimic. (B–F) Quantification of oxidative stress markers including GSH, GSSG, GSH/GSSG, NADPH, and MDA. (G) Representative immunofluorescence images of FerroOrange, ROS, and MitoSOX. Scale bar: 20 µm. (H) Immunofluorescence‐based lipid peroxidation staining. Scale bar: 20 µm. (I) Schematic of NPC treatment with siRNA and miRNA inhibitor: (1) si‐NC; (2) si‐*IL‐17A*; (3) miR‐548I inhibitor; (4) si‐*IL‐17A* + miR‐548I inhibitor. (J–N) Quantification of oxidative stress markers including GSH, GSSG, GSH/GSSG, NADPH, and MDA. (O) Immunofluorescence staining of FerroOrange, ROS, and MitoSOX. Scale bar: 20 µm. (P) Oxidative state staining. Scale bar: 20 µm. Data are presented as mean ± SD (*n* = 3). Statistical comparisons were performed using one‐way ANOVA with Tukey's post hoc test. *p < 0.05, **p < 0.01, ***p < 0.001, ****p < 0.0001, ns = not significant.

It has been reported that IL17A could regulate ferroptosis in various diseases, including allergic asthma and chronic cardiomyopathy [27, 33]. To investigate whether miR‐548I modulates ferroptosis through IL17A, we conducted rescue experiments by overexpressing IL17A (**Figure** **7**A). The coding sequence of IL17A was cloned into an expression vector lacking the miR‐548I binding site, thereby rendering it resistant to miRNA‐mediated repression. In human NPCs, transfection with miR‐548I mimics markedly enhanced intracellular antioxidant capacity, as indicated by increased GSH content, elevated GSH/GSSG ratio, and higher NADPH levels. These protective effects were significantly abolished by IL17A overexpression, which concomitantly restored intracellular GSSG and MDA levels (Figure 7B–F). Furthermore, miR‐548I mimics markedly reduced intracellular ROS, mitochondrial ROS, and Fe^2^^+^ accumulation, whereas IL17A overexpression partially reversed these changes (Figure 7G, Figure S10A–C). BODIPY fluorescence staining and quantification further confirmed that miR‐548I–mediated suppression of lipid peroxidation was substantially exacerbated by IL17A overexpression (Figure 7H, Figure S10D). In contrast, human NPCs transfected with miR‐548I inhibitor led to a marked reduction in intracellular GSH, GSH/GSSG ratio, and NADPH levels, accompanied by increased accumulation of GSSG and MDA (Figure 7I–N). Notably, silencing IL17A partially alleviated these oxidative stress alterations. Similarly, the elevated levels of intracellular Fe^2^^+^, ROS, and lipid peroxidation induced by miR‐548I inhibition were attenuated upon IL17A knockdown (Figure 7O,P, Figure S10E–H). Collectively, these results demonstrated that miR‐548I regulates ferroptosis by modulating the expression of IL17A.

### 2.10 Crispld2 Overexpression Alleviates the Degenerative Phenotype and Poor Pain‐Related Behavioral Scores Reversed by IL17A Overexpression

**FIGURE 8:**
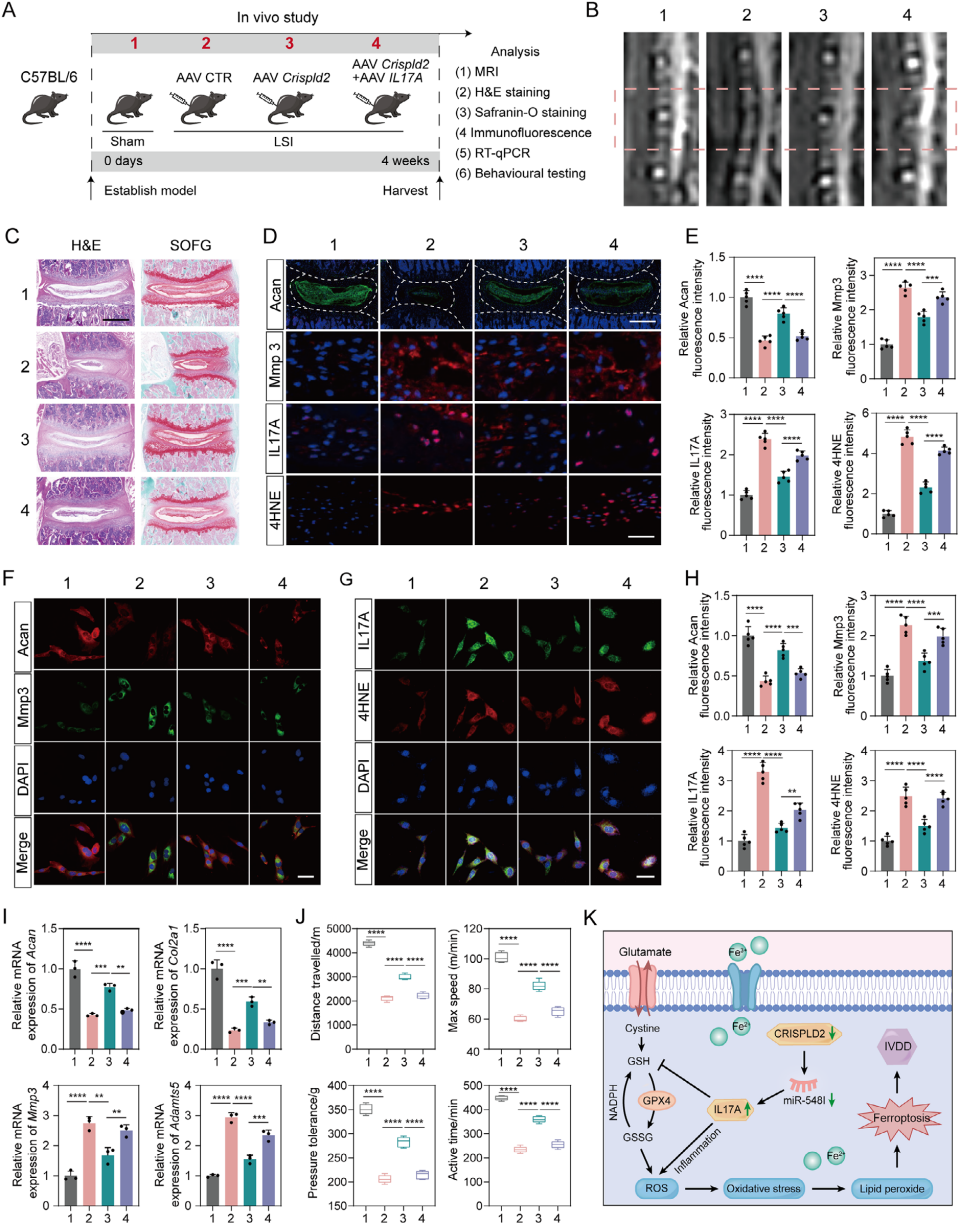
*Crispld2* overexpression alleviates disc degeneration and pain‐associated behaviors, which are reversed by *IL‐17A* overexpression in vivo. (A) Schematic timeline of LSI mouse model and AAV administration. (B) Representative MRI images of lumbar intervertebral discs in each group (*n* = 5). (C) H&E and SOFG staining in lumbar discs (*n* = 5). (D) Representative immunofluorescence images of disc sections stained for Acan, Mmp3, IL17A, and 4HNE. Scale bar: 500 µm (Acan), 50 µm (Mmp3, IL17A, and 4HNE), (*n* = 5). (E) Quantitative analysis of Acan, Mmp3, IL17A, and 4HNE protein expression (*n* = 5). (F,G) Immunofluorescence staining of primary NPCs isolated from mouse discs for Acan, Mmp3, IL17A, and 4HNE. Scale bar: 50 µm, (*n* = 5). (H) Quantification of relative fluorescence intensity for Acan, Mmp3, IL17A, and 4HNE in primary NPCs (*n* = 5). (I) RT‐qPCR analysis of mRNA expression of *Acan*, *Col2a1*, *Mmp3*, and *Adamts5* in NP tissues confirms molecular changes (*n* = 3). (J) Behavioral testing of mice, including distance traveled, maximum speed, pressure tolerance, and active time (*n* = 5). (K) Schematic summary: CRISPLD2 modulates disc degeneration via the miR‐548I‐IL17A axis to inhibit ferroptosis in NPCs. Data are presented as mean ± SD. Comparisons were performed using one‐way ANOVA with Tukey's post hoc test. *p < 0.05, **p < 0.01, ***p < 0.001, ****p < 0.0001, ns = not significant.

The results above demonstrated that *Crispld2* deficiency accelerates IVDD by promoting ferroptosis through IL17A upregulation, thereby exacerbating NPCs degeneration and pain‐related behavioral impairments. To further confirm the protective role of Crispld2, we established two in vivo IVDD models—LSI and CINS models—and delivered recombinant adeno‐associated viruses (AAVs) encoding *Crispld2* or *IL17A* into the NP region via intradiscal injection (Figure 8A, Figure S11A). MRI analysis revealed that discs in the control AAV group exhibited pronounced dehydration of the NP compared with the sham group. In contrast, *Crispld2* overexpression markedly alleviated these degenerative changes, whereas co‐administration of *Crispld2* and *IL17A* AAVs largely abrogated this protective effect (Figure 8B, Figure S11B). Histological staining, including H&E, SOFG, and Acan IF staining, confirmed the MRI results. *Crispld2* overexpression significantly increased proteoglycan content and reduced histological degeneration, while co‐expression of IL17A reversed these improvements (Figure 8C,E, Figure S11C,E). IF staining of disc sections further revealed that IL17A expression was significantly elevated in the degenerative discs of control AAV‐treated mice, whereas *Crispld2* overexpression markedly downregulated IL17A levels. This provides direct evidence that Crispld2 negatively regulates IL17A during disc degeneration. Moreover, IF staining demonstrated a significant reduction in Mmp3 and 4HNE (a marker of oxidative stress) secretion in NP tissues injected with *Crispld2* AAV compared to the control AAV group. Additionally, the simultaneous overexpression of Crispld2 and IL17A AAVs reversed this effect (Figure 8D,E, Figure S11D,E).

To validate these findings at the cellular level, primary NPCs were isolated from each group. IF analyses showed the relative expression of Acan, Mmp3, IL17A and 4HNE in NPCs was in line with the tissue‐level observations, reinforcing the idea that Crispld2 could influence disc cell homeostasis and oxidative stress responses through modulating IL17A (Figure 8F–H, Figure S11F–H). In addition, RT‐qPCR results demonstrated that *Crispld2* overexpression upregulated *Acan* and *Col2a1*, while downregulating *Mmp3* and *Adamts5*, which was reversed partly by the co‐administration of *Crispld2* and *IL17A* AAVs (Figure 8I, Figure S11I).

As pain is a prominent clinical manifestation of IVDD, and our previous findings indicated that *Crispld2*‐cKO mice exhibited spontaneous pain‐related behaviors during aging, we further assessed whether *Crispld2* overexpression could alleviate discogenic pain in the LSI model. Behavioral experiments revealed significantly poor pain‐related behavioral scores in control AAV‐treated mice, indicating marked hyperalgesia in the LSI model. Conversely, intrathecal administration of *Crispld2* AAV produced a significant reduction in pain sensitivity. Strikingly, combinatorial delivery of *Crispld2* and *IL17A* AAVs effectively counteracted the analgesic effect of *Crispld2* overexpression (Figure 8J). Collectively, these findings indicated that Crispld2 inhibits the degenerative phenotype of NPCs and mitigates IVDD progression and associated pain by regulating IL17A signaling. The underlying molecular mechanism is delineated in Figure 8K.

### 2.11 Crispld2 Deficiency Induces Discogenic Pain via Hyperalgesia and Enhanced Nociceptive Input

Pain is a major clinical manifestation of IVDD and significantly impairs quality of life. Increasing evidence suggests that neurotrophic factors and aberrant nerve ingrowth play key roles in the development of discogenic pain [34], with the expansion of nociceptive nerve fibers in degenerated discs serving as a critical anatomical basis for LBP [35]. In our study, analysis of clinical samples revealed a negative correlation between CRISPLD2 expression in NP tissue and patient‐reported pain severity. Moreover, *Crispld2‐cKO* mice showed more pronounced pain‐like behaviors. To explore whether the Crispld2 deficiency‐associated discogenic pain is associated with hyperalgesia and nerve growth, the mRNA levels of several indicators associated with neuroinflammation and nerve growth were detected, including cyclooxygenase 2 (*Cox2)*, nerve growth factor *(Ngf)*, and semaphorin 3A *(Sema3a*, a nerve regulator involved in inhibiting axonal growth). The results revealed that knockdown of *Crispld2* significantly alters the expression of these genes, with a notable increase in the levels of *Ngf* and *Cox2* and a remarkable decrease in the level of *Sema3a* (Figure S12A). The above results demonstrated that *Crispld2* downregulation potentially contributed to pain sensitivity in IVDD due to nerve growth and inflammation.

To further assess the interaction between NPCs and sensory neurons, particularly the effects of *Crispld2* knockdown on neuronal signaling, NPCs and dorsal root ganglion (DRG) sensory neurons were co‐cultured, and the schematic experimental setup was presented in Figure S12B. The morphology of DRG neurons was presented through NF200 immunofluorescence staining (Figure S12C). DRG neurons co‐cultured with *Crispld2‐*deficient NPCs displayed significantly longer neurite lengths at both 24 and 72 h compared to controls (Figure S12C,D), suggesting that Crispld2 was involved in inhibiting neurite outgrowth. Sholl analysis was performed to quantify the number of intersections in the neurite outgrowth of the cells [36], which revealed that co‐culture with NPCs from *Crispld2*‐cKO mice significantly increased the number of DRG neurons branching at both 24 and 72 h (Figure S12E).

Sensory neuropeptides involved in the pain‐transmitting pathway, such as substance P (SP) and calcitonin gene‐related peptide (CGRP), are positively associated with sensory sensitization in discogenic pain [37]. Therefore, we examined the effect of *Crispld2*‐deficient NP cells on DRG neuron sensitization by measuring the expression levels of these neuropeptides. RT‐qPCR analysis revealed that co‐culture with *Crispld2*‐deficient NPCs significantly upregulated *Cgrp* and *Sp* mRNA levels in DRG neurons (Figure S12F,G), which was further confirmed by immunofluorescence staining (Figure S12H,I). To validate these findings in vivo, we performed immunofluorescence analysis of DRG tissues. Following LSI surgery, *Crispld2^fl/fl^* mice showed markedly increased levels of Cgrp and Sp, indicating the activation of pain‐related pathways. Notably, both Sham and LSI groups of *Crispld2*‐cKO mice exhibited even higher expression levels of Cgrp and Sp compared to their *Crispld2^fl/fl^* counterparts (Figure S12J,K). Together, these results suggested that Crispld2 deficiency facilitates hyperalgesia, promotes nerve ingrowth, and enhances the activation of nociceptive transmission, thereby contributing to the development of discogenic pain.

## 3 Discussion

Current clinical treatments for IVDD primarily focus on symptomatic relief through pharmacological agents or surgical interventions. However, these approaches neither prevent disease progression nor restore the native structure and function of the intervertebral disc. Therefore, elucidating the molecular mechanisms underlying IVDD pathogenesis is essential for the development of novel and effective therapeutic strategies. In this study, we first identified CRISPLD2 as a key modulator of IVDD progression. Our findings reveal that reduced CRISPLD2 expression suppresses miR‐548I levels, resulting in the upregulation of IL17A, which in turn promotes oxidative stress–induced ferroptosis and accelerates disc degeneration. Critically, this mechanistic insight provides a potential foundation for translational intervention. Gene therapy approaches aimed at restoring CRISPLD2 expression may provide a novel strategy to mitigate disc degeneration and preserve NPCs function. Furthermore, considering the pro‐inflammatory and ferroptosis‐promoting effects of IL17A, combining CRISPLD2‐based therapy with existing IL17A inhibitors (e.g., Secukinumab) could further enhance therapeutic efficacy.

Interestingly, analysis of both clinical samples and single‐cell transcriptomic data revealed a non‐linear expression pattern of CRISPLD2 during IVDD progression. CRISPLD2 expression exhibited a transient increase at mild degeneration stages (NPC3 and Pfirrmann grade III), followed by a pronounced decline in more advanced degeneration (NPC4 and Pfirrmann grades IV–V). This ‘rise‐then‐fall’ pattern likely reflects an early compensatory response, where CRISPLD2, known to possess anti‐inflammatory and protective functions, may be upregulated to counteract initial inflammatory and oxidative stress stimuli. However, with sustained degeneration, prolonged inflammation, and mitochondrial dysfunction, this compensatory mechanism appears to collapse, resulting in reduced CRISPLD2 expression. This dynamic regulation is further supported by our IL‐1β gradient experiments, in which low‐dose or short‐term stimulation elevated CRISPLD2 expression, whereas higher‐dose or prolonged exposure suppressed it, mimicking progressive degeneration in vitro. These insights emphasize the importance of CRISPLD2 as a dynamically regulated protective mediator and suggest that interventions targeting its decline may hold therapeutic value in halting IVDD progression.

Ferroptosis, a regulated form of cell death characterized by iron accumulation and lipid peroxidation, has been increasingly recognized in the pathogenesis of IVDD [14, 16, 38]. Our study provides the first direct evidence linking CRISPLD2 to ferroptosis in IVDD. Specifically, CRISPLD2 knockdown led to the upregulation of ferroptosis‐related genes and the accumulation of lipid peroxides, indicating that loss of CRISPLD2 shifts the redox balance toward a ferroptosis‐prone state. Oxidative stress, characterized by excessive production of ROS, serves as a major upstream driver of ferroptosis by disrupting redox homeostasis and inactivating key antioxidant enzymes. This oxidative environment facilitates the accumulation of toxic lipid hydroperoxides, amplifying ferroptotic cell death. The pathological crosstalk between oxidative stress and ferroptosis has been demonstrated in neurodegenerative and musculoskeletal diseases, including Alzheimer's disease and ischemic stroke [17, 18]. Our findings aligned with and extended these observations by showing that CRISPLD2 deficiency exacerbates both oxidative stress and ferroptosis in NPCs, resulting in enhanced cell loss and extracellular matrix degradation. This highlights CRISPLD2 as a novel regulator of oxidative stress–induced ferroptosis in disc degeneration. Targeting this pathway may offer new therapeutic opportunities for halting or reversing the progression of IVDD.

Previous studies have revealed that inflammation is another key driver of IVDD progression, with pro‐inflammatory cytokines such as IL‐1β, TNF‐α, and IL17A playing pivotal roles in disc degradation [39, 40]. Our study revealed that CRISPLD2 downregulation leads to increased IL17A expression, which in turn exacerbates ferroptosis and inflammation in NPCs. IL17A had been extensively studied in inflammatory diseases, including rheumatoid arthritis and osteoarthritis, where it enhances inflammatory cascades and degrades extracellular matrix components [41, 42]. In IVDD, IL17A had been shown to promote catabolic processes through increasing MMP activity [43]. Our study further elucidated its role in IVDD by demonstrating that IL17A not only drives inflammation but also facilitates ferroptosis, leading to greater disc degeneration. The regulatory effect of CRISPLD2 on IL17A expression suggested a protective mechanism against IL17A‐mediated inflammation and oxidative stress. Moreover, recent research has identified IL17A inhibitors as promising therapeutic agents in musculoskeletal diseases [44, 45], and our study provided additional evidence supporting IL17A as a potential target for IVDD treatment.

Our findings also established a novel miR‐548I‐IL17A regulatory axis controlled by CRISPLD2 in IVDD. MicroRNAs have emerged as critical post‐transcriptional regulators of gene expression in degenerative diseases, and their roles in ferroptosis and inflammation are becoming increasingly evident [46]. Recent studies have identified several miRNAs, including miR‐522 and miR‐1443p, as regulators of ferroptosis by targeting genes involved in iron metabolism and lipid peroxidation [47, 48]. In our study, CRISPLD2 deficiency led to a reduction in miR‐548I expression, which subsequently increased IL17A levels and promoted ferroptosis in NPCs. This suggested that miR‐548I acts as a protective factor by suppressing IL17A‐induced oxidative stress. Given that miRNA‐based therapies are being actively explored for various inflammatory and degenerative diseases, targeting the CRISPLD2‐miR‐548I‐IL17A axis could offer a novel therapeutic approach for IVDD. It is worth noting that although our results demonstrate that CRISPLD2 regulates IL17A expression through miR‐548I, the upstream mechanism by which CRISPLD2 modulates miR‐548I itself remains unclear. Several possibilities merit consideration. CRISPLD2 may activate membrane‐associated receptors and thereby influence intracellular signaling pathways that enhance miR‐548I transcription. Alternatively, CRISPLD2 could modulate miRNA maturation or stability at the post‐transcriptional level by affecting components of the miRNA‐processing machinery or RNA‐binding proteins involved in miRNA turnover. While these explanations remain speculative, they highlight the novelty of this regulatory relationship and provide important directions for future mechanistic studies.

Our present study also revealed that CRISPLD2 deficiency contributes to discogenic low back pain in IVDD by regulating nerve growth and pain perception. Emerging evidence suggested that sensory and autonomic nerve fibers infiltrate degenerative IVDs, correlating with the progression of discogenic pain [49]. The sensory innervation of the IVD originates primarily from the sinuvertebral nerve and dorsal root ganglion (DRG), playing a pivotal role in pain transmission [50, 51, 52, 53]. Sympathetic nerve fibers also modulate inflammation and ECM remodeling [54]. Moreover, pro‐inflammatory cytokines and ECM‐degrading enzymes create a feedback loop that promotes neuroinflammation and accelerates disc degeneration. Our previous studies revealed that degenerative discs exhibit altered expression of neurotrophic factors such as CGRP, vasoactive intestinal peptide (VIP), and neuropeptide Y (NPY) [55, 56, 57], which play important roles in regulating nerve infiltration and sensitization of nociceptive neurons. In this present study, CRISPLD2 depletion led to heightened pain sensitivity and promoted axonal growth of dorsal root ganglion neurons, indicating that CRISPLD2 plays a role in regulating neuroinflammation and pain signaling. This aligned with research showing that inflammation‐induced nerve growth exacerbates pain perception in IVDD [34, 58]. The dual role of CRISPLD2 in preventing both structural degeneration and pain hypersensitivity suggested that it may be a valuable therapeutic target for managing both IVDD progression and associated pain.

Our findings open several promising and feasible avenues for the clinical translation of therapies targeting the CRISPLD2 pathway for IVDD. One direct strategy is the further development of AAV‐mediated CRISPLD2 gene therapy, similar to the approach validated in our study. Given the contained nature of the intervertebral disc, a single intradiscal injection could provide long‐term, localized protein expression, potentially offering a one‐time treatment to halt degeneration and alleviate pain. Future work must focus on optimizing AAV serotypes for efficient transduction of human nucleus pulposus cells and conducting comprehensive long‐term safety assessments. An alternative and potentially safer non‐viral strategy would involve the intradiscal administration of synthetic miR‐548I mimics. This approach would bypass the need for gene delivery and directly replenish the downstream effector molecule. The primary challenges for miRNA‐based therapies include ensuring stability in the harsh disc microenvironment and achieving efficient cellular uptake. Encapsulating miR‐548I mimics in biocompatible carriers, such as hydrogels or lipid nanoparticles, could enhance their local retention and therapeutic efficacy.

Despite the novel insights provided by our study, several limitations should be acknowledged. First, the number of clinical samples included in this study was relatively small, and larger cohorts are needed to validate and extend our findings. Second, although we identified a strong association between CRISPLD2 expression and ferroptosis regulation, it remains unclear whether CRISPLD2 also influences other forms of cell death and further mechanistic studies are warranted to explore this possibility. Third, while we confirmed our results in both in vitro and in vivo models, the inherent differences between animal models and human IVDD may limit the direct translational applicability of our findings. Future preclinical studies should aim to validate these findings in large animal models, whose disc biology more closely resembles that of humans. Fourth, the precise mechanism by which CRISPLD2 regulates miR‐548I, whether through transcriptional, epigenetic, or post‐transcriptional pathways, was not investigated and requires further clarification. Fifth, while we demonstrated that CRISPLD2 deficiency promotes both NPC ferroptosis and pain‐like behaviors, we did not directly test whether specific inhibition of ferroptosis in vivo could ameliorate hyperalgesia. The precise molecular link between ferroptotic disc cells and sensory neuron sensitization remains a critical question for future investigation. Finally, while our AAV‐based gene therapy showed promising results, its path to the clinic requires further research to optimize delivery efficiency and conduct rigorous, long‐term toxicology studies. Systemic administration of CRISPLD2 poses a risk of off‐target effects due to its critical roles in other tissues, like the lungs, and its involvement in the innate immune response. This necessitates the development of targeted local delivery strategies for future therapeutic applications.

## 4 Conclusion

In conclusion, our study identifies CRISPLD2 as a critical regulator of IVDD progression through its modulation of oxidative stress‐induced ferroptosis via the miR‐548I‐IL‐17A axis. By maintaining NPC homeostasis and preventing oxidative stress‐induced cell death, CRISPLD2 plays a protective role in preserving intervertebral disc integrity. Additionally, its influence on neuroinflammation and pain sensitivity highlights its potential as a dual therapeutic target for both disc degeneration and discogenic pain. These findings provide valuable insights into the molecular mechanisms of IVDD and suggest that targeting CRISPLD2 may offer new strategies for therapeutic intervention in degenerative disc disease.

## 5 Experimental Section

### 5.1 Human NP Tissues Collection

Human nucleus pulposus tissues were collected from 43 patients undergoing discectomy due to spinal deformity, tethered cord syndrome, or IVDD (21 female, mean age = 50.0 ± 14.3 years; 22 men, mean age = 49.5 ± 13.1 years). None of the participants had endocrine abnormalities, neurologic disorders, or autoimmune diseases such as hyperthyroidism, ankylosing spondylitis, rheumatoid arthritis, adrenal insufficiency, or systemic lupus erythematosus. The severity of IVDD was evaluated based on MRI using the Pfirrmann grading system by two independent spine surgeons who were blinded to the clinical data. Samples with Pfirrmann grade II were classified as the normal group (*n* = 7, mean age = 31.0 ± 5.9 years), while those with graded III (*n* = 11, mean age = 46.8 ±10.4 years), IV (*n* = 14, mean age = 55.1 ± 10.8 years), or V (*n* = 11, mean age = 57.8 ± 11.0 years) were categorized and assigned to the IVDD group. Detailed sample information is provided in Table S1. This study was approved by the Ethics Committee of our Hospital (NMC2023018‐008), and written informed consent was obtained from all participants. All procedures involving human NP tissues were conducted in accordance with the Declaration of Helsinki.

### 5.2 Isolation and Culture of Human NPCs

All surgically excised nucleus pulposus tissues were transported and processed under sterile conditions. After washing with PBS, the tissues were finely minced and sequentially digested with 0.25% trypsin‐EDTA (G4001, Servicebio, China) for 30 min, followed by 0.2% collagenase type II (A004174‐0100, Sangon Biotech, China) for 60 min in a 37°C water bath. The resulting cell suspension was passed through a 70 µm cell strainer and centrifuged at 1200 rpm for 5 min. The cell pellet was then resuspended in DMEM/F‐12 (11330057, Gibco, Australia) medium supplemented with 15% fetal bovine serum (10099‐141C, Gibco, Australia) and 1% penicillin‐streptomycin (15140163, Gibco, Australia). Cells were cultured at 37°C in a humidified incubator with 5% CO_2_ and expanded until reaching 80%–90% confluence, with medium changes every 2–3 days. To ensure the stability of cellular phenotype and avoid phenotypic drift, all experiments in this study were performed using human NPCs between passages 2 and 4.

### 5.3 Western Blot

Samples were homogenized in ice‐cold RIPA buffer containing protease and phosphatase inhibitors. The protein concentrations were determined using the BCA protein assay kit (P0010, Beyotime, China). Equal amounts of protein (30 µg) were loaded onto 10% SDS‐PAGE gels and separated by electrophoresis. After electrophoresis, proteins were transferred to a polyvinylidene fluoride (PVDF) membrane (IPVH00010, Millipore, USA) and blocked with 5% non‐fat dry milk in Tris‐buffered saline with 0.1% Tween‐20 (TBST) for 1 h at room temperature. The membrane was then incubated overnight at 4°C with primary antibodies diluted in TBST. The primary antibodies used were as follows: anti‐Crispld2 (DF14963, Affinity, China, 1:1000), anti‐ACAN (DF7561, Affinity, China, 1:1000), anti‐COL2A1 (AF0135, Affinity, China, 1:1000), anti‐MMP3 (AF0217, Affinity, China, 1:1000), anti‐ADAMTS5 (DF13268, Affinity, China, 1:1000), anti‐IL‐17A (GB150079‐100, ServiceBio, China, 1:1000), anti‐xCT (DF12509, Affinity, China, 1:1000), anti‐ACSL4 (F12141, Affinity, China, 1:1000), anti‐FTH1 (381204, ZenBioScience, China, 1:1000), anti‐GPX4 (R24461, ZenBioScience, China, 1:1000), anti‐4HNE (bs‐6313R, Bioss, China, 1:1000), and anti‐β‐actin (GB15003‐100, ServiceBio, China, 1:3000) as a loading control. After washing with TBST, the membrane was incubated with the corresponding horseradish peroxidase (HRP)‐conjugated secondary antibody (ServiceBio, China, GB23303, GB23301, 1:5000) for 1 h at room temperature. Protein bands were visualized using the ECL Kit (SQ201L, Epizyme Biomedical Technology Co., Ltd, China), and the images were captured using an automatic chemiluminescence/fluorescence image analysis system (5200, Tanon, China). Densitometric analysis of the protein bands was performed using ImageJ software (National Institutes of Health).

### 5.4 RNA Extraction and Quantitative Real‐Time PCR

Total RNA was extracted from samples using the Trizol reagent according to the manufacturer's instructions (R4013‐02, Magen, China). The concentration and quality of the RNA were assessed using a NanoDrop spectrophotometer. Complementary DNA (cDNA) was synthesized from 1 µg of total RNA using the PrimeScript RT reagent Kit according to the manufacturer's protocol (R323‐01; Vazyme, China). RT‐qPCR was performed using the SYBR Green Master Mix (Q711‐03, Vazyme, China). The amplification protocol consisted of an initial denaturation at 95°C for 10 min, followed by 40 cycles of 95°C for 15 s and 60°C for 1 min. A dissociation curve was generated to confirm the specificity of amplification. The relative gene expression was calculated using the 2^−△△Ct^ method, with β‐actin used as the internal control. The primers used in the RT‐qPCR analysis were listed in Table S2.

### 5.5 Immunohistochemistry

IHC staining was performed to assess the expression and localization of ACAN in tissue sections following a standardized protocol. Paraffin‐embedded tissue samples were sectioned at 4‐µm thickness using a microtome. The sections were deparaffinized in xylene and rehydrated through a graded ethanol series before being subjected to antigen retrieval using 10 mM citrate buffer (pH 6.0) at 95°C for 15 min in a pressure cooker. Endogenous peroxidase activity was quenched by incubating sections in 3% hydrogen peroxide (H_2_O_2_) for 10 min at room temperature. After blocking nonspecific binding with 5% bovine serum albumin (BSA) in PBS for 30 min, tissue sections were incubated with the primary antibody anti‐ACAN (DF7561, Affinity, China, 1:200) overnight at 4°C in a humidified chamber. The next day, sections were washed three times in PBS and incubated with a horseradish peroxidase (HRP)‐conjugated secondary antibody (511203, ZenBioScience, China, 1:500) for 1 h at room temperature. Signal detection was performed using the 3,3'‐diaminobenzidine (DAB) substrate kit (G1212, ServiceBio, China), followed by counterstaining with hematoxylin. After dehydration in graded ethanol and xylene, slides were mounted with neutral resin and visualized under a light microscope. The immunoreactivity of ACAN was evaluated based on staining intensity and the percentage of positive cells. Quantification was performed using ImageJ software (NIH, USA).

### 5.6 Immunofluorescence of Cells and Tissue Sections

IF staining was performed on both cultured cells and tissue sections to assess the expression and localization of specific target proteins. Cells were seeded onto glass coverslips, fixed with 4% paraformaldehyde for 15 min at room temperature, and permeabilized using 0.1% Triton X‐100 in phosphate‐buffered saline (PBS) for 10 min. Tissue sections were deparaffinized, rehydrated through graded ethanol solutions, and subjected to antigen retrieval using citrate buffer (pH 6.0) at 95°C for 15 min. Blocking was conducted with 5% bovine serum albumin (BSA) for 1 h at room temperature to prevent non‐specific binding. Samples were incubated overnight at 4°C with primary antibodies, followed by three washes with PBS. Secondary antibodies were applied for 1 h at room temperature in the dark. Nuclei were counterstained with 4′,6‐diamidino‐2‐phenylindole (DAPI) for 5 min. Fluorescent signals were visualized using a confocal scanning microscope (Leica SP8), and images were processed with ImageJ software (NIH). The primary antibodies used in this study included Crispld2 (DF14963, Affinity, China, 1:200), ACAN (DF7561, Affinity, China, 1:100), MMP3 (AF0217, Affinity, China, 1:200), IL‐17A (GB11110‐1‐100, ServiceBio, China, 1:200), GPX4 (R24461, ZenBioScience, China, 1:100), FTH1 (381204, ZenBioScience, China, 1:200), ACSL4 (F12141, Affinity, China, 1:100), CGRP (ab81887, Abcam, 1:200) and SP (DF7522, Affinity, China, 1:200).

### 5.7 Hematoxylin and Eosin and Safranin O & Fast Green staining

H&E and SOFG staining were performed on intervertebral disc tissue sections to evaluate histological structure and proteoglycan distribution. For HE staining, sections were deparaffinized in xylene, rehydrated through a graded ethanol series, and stained with Harris hematoxylin for 5 min, followed by differentiation in 1% acid alcohol and counterstaining with eosin for 2 min. For SOFG staining, sections were stained with 0.1% Fast Green for 5 min, differentiated in 1% acetic acid for 10 s, and counterstained with 0.1% Safranin O for 5 min. Slides were dehydrated, cleared in xylene, and mounted with neutral resin. Images were captured using a light microscope, and histological scoring was performed using established grading criteria to assess intervertebral disc degeneration.

### 5.8 Construction of Gene Conditional Knockout Mice

All animal studies were performed in accordance with protocols approved by the Institutional Animal Care and Use Committee of Naval Medical University, and adhered to institutional and national guidelines for animal experiments. *Col2a1*‐*Cre* transgenic mice on a C57BL/6J background and *Crispld2^fl/fl^* mice on a C57BL/6 background were provided by GemPharmatech Co., Ltd. These mice were crossed to generate NP‐specific *Crispld2* conditional knockout (cKO) mice (*Col2a1‐Cre*; *Crispld2^fl/fl^*) as described previously. All animals were housed under specific pathogen‐free conditions at room temperature (23 ± 2°C) with a 12 h light/dark cycle and ad libitum access to food and water.

### 5.9 Construction of a Mouse Model of IVDD

The surgical technique of lumbar spine instability (LSI) model followed the protocol described in our previous study [19]. Under general anesthesia with 2% isoflurane, a posterior midline skin incision was made to expose the lumbar spine. Bilateral paraspinal muscles were carefully separated to access the L3‐L5 vertebral levels. Instability was induced by surgically transecting the posterior spinal structures, including the interspinous and supraspinous ligaments, while preserving the vertebral body and intervertebral discs to mimic pathological lumbar instability. The surgical site was irrigated with sterile saline, and the incision was closed in layers with absorbable sutures. Mice were housed under standard laboratory conditions with ad libitum access to food and water. Sham‐operated mice, in which the posterior elements were exposed but not transected, served as controls.

The coccygeal IVDDs needle stab (CINS) model was established in the tail discs (caudal vertebrae, Co6/7) of the mice [59]. Mice were anesthetized with 1%–2% isoflurane inhalation and placed in a prone position. The target disc was located by palpation, and the overlying skin was sterilized with 70% ethanol and povidone‐iodine solution. A 26‐gauge sterile needle was inserted perpendicularly into the disc space through the tail skin, penetrating the AF and NP tissues to a depth of approximately 1.5 mm. The needle was rotated 180° and held in place for 30 s before being carefully withdrawn. Sham‐operated mice underwent the same procedure without needle insertion. After the procedure, the mice were observed for signs of distress, pain, or infection. All the animals were euthanized at 4 weeks post‐surgery for sample collection and subsequent analysis of IVDD.

### 5.10 Reactive Oxygen Species Analysis

The ROS levels in NPCs were measured using the ROS detection kit (S0035S, Beyotime, China) following the manufacturer's instructions. Fluorescence intensities were quantified and normalized to the untreated control group. All experiments were performed in triplicate.

### 5.11 Mitochondrial Superoxide Assay

Mitochondrial superoxide in NPCs was assessed using the MitoSOX Red probe (M36008; Invitrogen, USA) according to the manufacturer's protocol. Briefly, adherent cells were washed with PBS, incubated with 1 mL MitoSOX Red working solution per well of a six‐well plate at 37°C for 30 min, and then rinsed twice with PBS to remove residual dye. Fluorescence was subsequently visualized using a confocal scanning microscope.

### 5.12 Determination of Fe^2^^+^ in Cells

The intracellular labile ferrous iron (Fe^2^^+^) levels in NPCs were detected using the FerroOrange fluorescent probe following the manufacturer's instructions. Cells were analyzed using a confocal scanning microscope. All experiments were conducted in triplicate.

### 5.13 Lipid Peroxidation Assay

Lipid peroxidation was assessed using the fluorescent probe BODIPY 581/591 C11 (D3861; Invitrogen) following the manufacturer's protocol. NPCs were incubated with 2 µM probe for 1 h, washed twice with ice‐cold sterile PBS, and subsequently fixed in 4% paraformaldehyde. After mounting with an anti‐fade reagent (G1401, Servicebio, China), lipid peroxidation was quantified as the ratio of green to red fluorescence intensity.

### 5.14 GSH/ GSSG Ratio Measurement

The intracellular levels of reduced GSH, GSSG, and the GSH/GSSG ratio in NPCs were quantified using a commercial GSH and GSSG detection kit (S0053, Beyotime, China) following the manufacturer's instructions. Each experiment was performed in triplicate.

### 5.15 Nicotinamide Adenine Dinucleotide Phosphate Assay

The NADPH in NPCs was measured using the enhanced NADP+/NADPH assay kit via WST‐8 method (S0179, Beyotime, China) following the manufacturer's protocol. All experiments were performed in triplicate.

### 5.16 Malondialdehyde Assay

The levels of MDA, a key marker of lipid peroxidation, in NPCs were measured using the lipid peroxidation MDA assay kit (S0131S, Beyotime, China) following the manufacturer's instructions. The absorbance of the supernatant was measured at 532 nm using a microplate reader (BioTek, USA). Each experiment was performed in triplicate.

### 5.17 Transmission Electron Microscopy for NPCs

When cell confluence reached 80%, the culture medium was removed and cells were rinsed three times with cold PBS. Cells were digested with 0.25% trypsin (G4001, Servicebio, China) for 3 min and collected by centrifugation at 1500 rpm for 10 min. The pellet was fixed in 2.5% glutaraldehyde (G1102, Servicebio, China), dehydrated through a graded ethanol series (20 min each), and treated twice with acetone (15 min each). Resin infiltration and embedding were performed as follows: acetone: epoxy resin (1:1) for 2–4 h at 37°C, then acetone: epoxy resin (1:2) overnight at 37°C. Samples were transferred into pure epoxy resin in embedding molds and kept at 37°C overnight, followed by polymerization at 65°C for >48 h. The resulting resin blocks were removed from molds and stored at room temperature until use. Ultrathin sections (∼75 nm) were cut with an ultramicrotome, stained with 2% uranyl acetate (8 min, light‐protected) and 2.6% lead citrate (8 min, CO_2_‐free), and imaged with a transmission electron microscope (HT7800, HITACHI, Japan).

### 5.18 Dual‐Luciferase Assay

To investigate the regulatory effect of hsa‐miR‐548I on IL17A, a dual‐luciferase reporter assay was performed using the HEK293T cell line. The full‐length 3' untranslated region (UTR) of IL17A was cloned into the pmirGLO dual‐luciferase vector (Promega), which contains both firefly and Renilla luciferase reporter genes. The pmirGLO‐IL17A‐3'UTR plasmid was co‐transfected with either the hsa‐miR‐548I mimic or negative control (NC) into HEK293T cells using Lipofectamine 3000 (Invitrogen) according to the manufacturer's protocol. After 48 h of transfection, luciferase activity was measured using the GLO‐MAX 20/20 luminometer.

### 5.19 miRNA Inhibitor and Mimics Transient Infection

MiRNA inhibitors and mimics were purchased from RiboBio company. According to the manufacturer’ s instructions, when NP cells were cultured at 60−70% density, miRNA inhibitors or mimics were transfected into NP cells via lipofectamine 3000 (Invitrogen, L3000008). After being transfected for 1 day, the transfection medium was removed.

### 5.20 Untargeted Lipidomics Analysis

NPCs were seeded in 6‐well plates at a density of 1 × 10^7^ cells per well and transfected with si‐NC, si‐*CRISPLD2* or a combination of si‐*CRISPLD2* and si‐*IL17A* using Lip3000 for 4 h. After treatment, cells were harvested and stored at –80°C until analysis. Lipid extraction was carried out by Cosmos Wisdom Laboratory (Cosmos Wisdom Co., Ltd., Hangzhou). The extracted lipids were separated using high‐performance liquid chromatography (HPLC) equipped with a Thermo Accucore C18 column (2.1 mm × 100 mm, 2.6 µm). Mass spectrometry (MS/MS) was performed on a Q‐Exactive hybrid quadrupole‐Orbitrap system (Thermo Fisher Scientific) with the following operating parameters: IS 5200/–4500, TEM 500, CUR 35, GS1 55, GS2 55. Lipid identification, functional annotation, and classification were based on the LIPID MAPS Lipidomics Gateway (https://www.lipidmaps.org/) and the LION (Lipid Ontology) database (http://lipidontology.com/). Data visualization was performed using the R package ggplot2 (version 3.4.4). Differential lipids were defined as those with a *p <* 0.05 and a variable importance in projection (VIP) score > 1.0.

### 5.21 Transcriptome Sequencing and Bioinformatics analysis

Human NPCs were seeded in 6‐well plates (1 × 10^5^ cells/well) and cultured for 24 h to ensure adherence, followed by transfection with si‐NC or si‐*CRISPLD2* for another 24 h. Total RNA was then extracted using TRIZOL reagent and stored at −80°C. mRNA sequencing was carried out in collaboration with Genekinder Medicaltech (China). Differentially expressed genes (DEGs) were identified with DESeq2 using the thresholds |log2FC| > 1 and FDR < 0.05 [59], and results were visualized through volcano plots (ggplot2) and heatmaps (heatmap package).

### 5.22 Magnetic Resonance Imaging

T2‐weighted MRI was conducted using a 3.0 Tesla scanner (United Imaging, China) to evaluate morphological and signal intensity alterations in the IVD of mice. Higher T2 signal intensity was indicative of healthier disc tissue. The degree of disc degeneration in mice was evaluated based on T2‐weighted images using the Pfirrmann grading system, which classifies disc degeneration into five grades, from Grade I (normal) to Grade V (severe degeneration).

### 5.23 Behavior Testing

Four distinct behavioral tests related to pain were conducted. To avoid subjective bias, all behavioral assessment were performed by an experimenter who was blinded to the group assignments. To assess pressure hyperalgesia, vocalization thresholds were measured using a force gauge (Bioseb, USA). Mice were restrained, and the sensor tip was applied to the skin over the L4/5 region with a gradually increasing force of 50 g/s until vocalization occurred. Additionally, three parameters of spontaneous activity—distance traveled, total active duration, and peak speed—were monitored using a wheel activity apparatus (Bioseb, USA). The device allowed mice to spin the wheel in both directions, and the data were automatically recorded and analyzed by an operator who remained blinded to the treatment groups throughout the experiment.

### 5.24 Small Interfering RNA Transfection

To silence human *CRISPLD2* or *IL17A*, small interfering RNA (siRNA) sequences were designed in collaboration with GenePharma and Genomeditech, respectively (Shanghai, China). Human NPCs were seeded into 12‐well plates, and once cell density reached 60%–70%, siRNA transfection was carried out using Lipofectamine 3000 (Invitrogen) according to the manufacturer's instructions. The synthetic siRNA sequences are listed in Table S3. After 24 h, total RNA was extracted and RT‐qPCR was performed to evaluate knockdown efficiency. Ultimately, CRISPLD2‐si‐4, and IL17A‐si‐1 were identified as the most effective siRNAs (Figure S13). The optimal sequence for human *CRISPLD2* was CAAGUCGUCAGAUGUGACATT (sense) and UGUCACAUCUGACGACUUGTT (antisense), while that for human *IL17A* was GGUCCUCAGAUUACUACAACC (sense) and UUGUAGUAAUCUGAGGACCUU (antisense).

### 5.25 Plasmid Transfection

The coding sequences (CDS) of CRISPLD2 (GenBank ID: M679414.1) and IL17A (GenBank ID: OM794745.1) were retrieved from the National Center for Biotechnology Information (https://www.ncbi.nlm.nih.gov/). Both full‐length sequences were synthesized and cloned into a Flag vector by Genemeditech Biotechnology (Shanghai, China). Human NPCs were transfected with the recombinant plasmids using Lipo8000 reagent (C0533, Beyotime) according to the manufacturer's protocol. After 24–48 h of incubation, cells were harvested for mRNA and protein extraction for subsequent experiments.

### 5.26 Adeno‐Associated Virus Infection

To enhance the in vivo expression of Crispld2 or IL17A, recombinant AAV9 vectors carrying the respective genes were constructed, while AAV9‐NC served as the control (Genemeditech, Shanghai, China). Following model establishment, viral particles (1 × 10^1^ ^2^ GC/mL, 1 µL) were delivered into the IVD tissue using a 5 µL Hamilton microsyringe (MICROLITER Series; Hamilton Bonaduz, Switzerland). After injection, the needle was withdrawn carefully to minimize mechanical injury to the NP tissue.

### 5.27 Statistical Analysis

All experiments in this study were independently conducted at least three times. Data are expressed as mean ± standard deviation (SD). Statistical analyses were performed using GraphPad Prism 9 (GraphPad Software Inc., La Jolla, CA, USA). Prior to statistical comparisons, the normality and homogeneity of variance were assessed using Shapiro's test and Levene's test, respectively. Group comparisons were carried out using two‐tailed unpaired Student's *t*‐tests or one‐way analysis of variance (ANOVA), followed by Tukey's post hoc test. A *p*‐value less than 0.05 was considered statistically significant (*p < 0.05, **p < 0.01, ***p < 0.001, ****p < 0.0001).

### Ethics Statement

The use of human tissues was approved by the Institutional Human Ethics Review Board of Changzheng Hospital, with approval number SL2023011‐A. In addition, authorization to conduct experiments involving animal subjects was granted by the Institutional Human Ethics Review Board of Changzheng Hospital, with approval number SL2023011‐B.

## Conflicts of Interest

The authors declare no conflict of interest.

## Supporting information




**Supporting File**: advs73723‐sup‐0001‐SuppMat.docx.

## Data Availability

The data that support the findings of this study are available from the corresponding author upon reasonable request.
