# Supplementary material for: CRISPLD2 Attenuates Intervertebral Disc Degeneration by Suppressing Oxidative Stress‐Induced Ferroptosis through the miR‐548I‐IL17A Axis
Source: Adv Sci (Weinh). 2026 Jan 9;13(16):e16477. doi: 10.1002/advs.202516477 (PMC13042808; doi:10.1002/advs.202516477)
Supplement: Supplementary file 1 — Supporting File: advs73723‐sup‐0001‐SuppMat.docx. [file ADVS-13-e16477-s001.docx]

Supplementary Materials for

CRISPLD2 Attenuates Intervertebral Disc Degeneration by Suppressing Oxidative Stress-Induced Ferroptosis through the miR-548I-IL17A Axis

**This PDF file includes:**

Fig. S1-13

Table S1-3

**
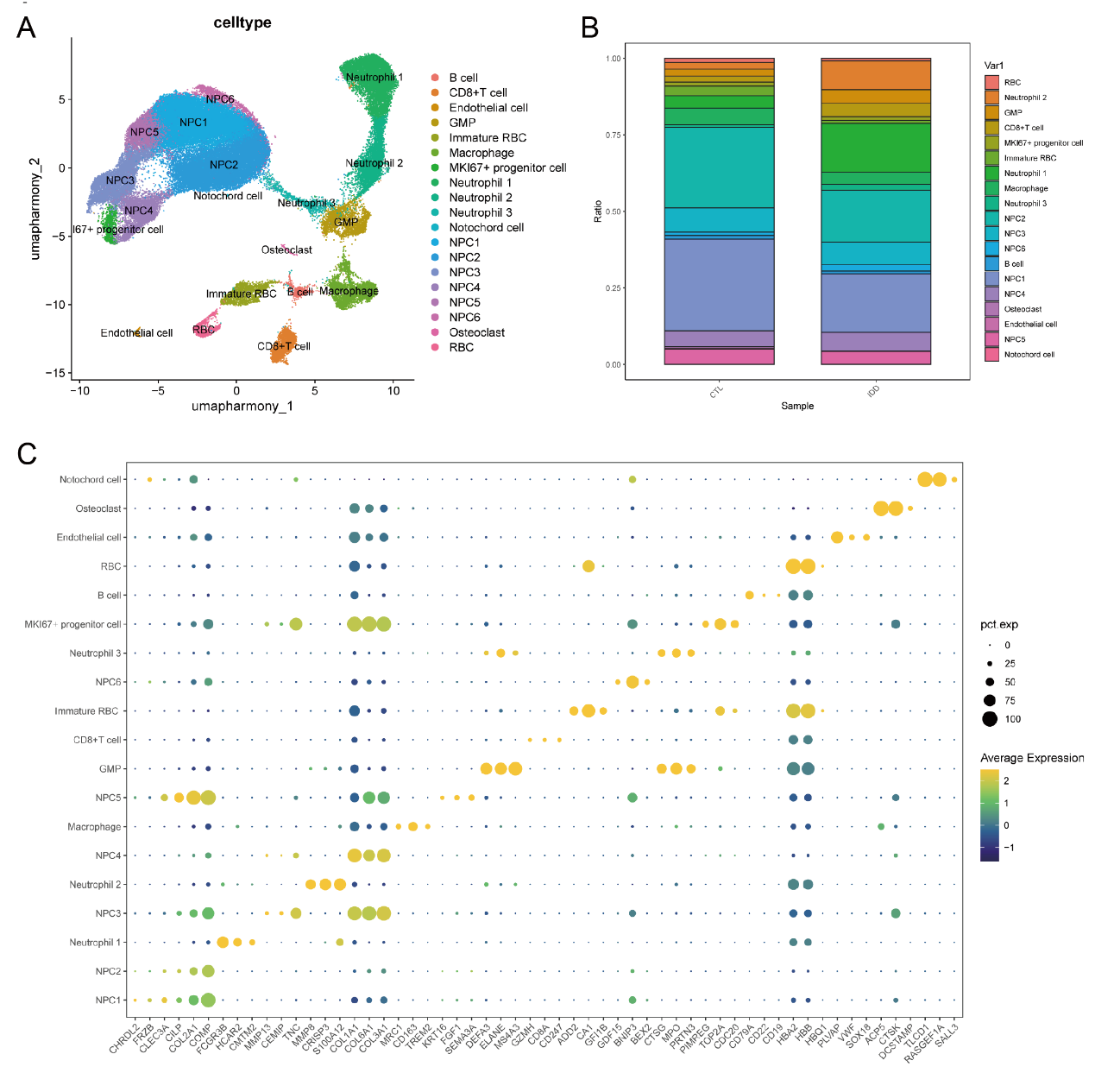
**

**Supplementary figure S1**. Single-cell transcriptomic analysis reveals cellular heterogeneity and ferroptosis-associated NPC subtypes in IDD. UMAP visualization of single-cell transcriptomes from intervertebral disc tissues identifies multiple cell populations. (B) Proportion of cell populations in CTL and IDD groups. (C) Expression distribution of genes across various cell types.


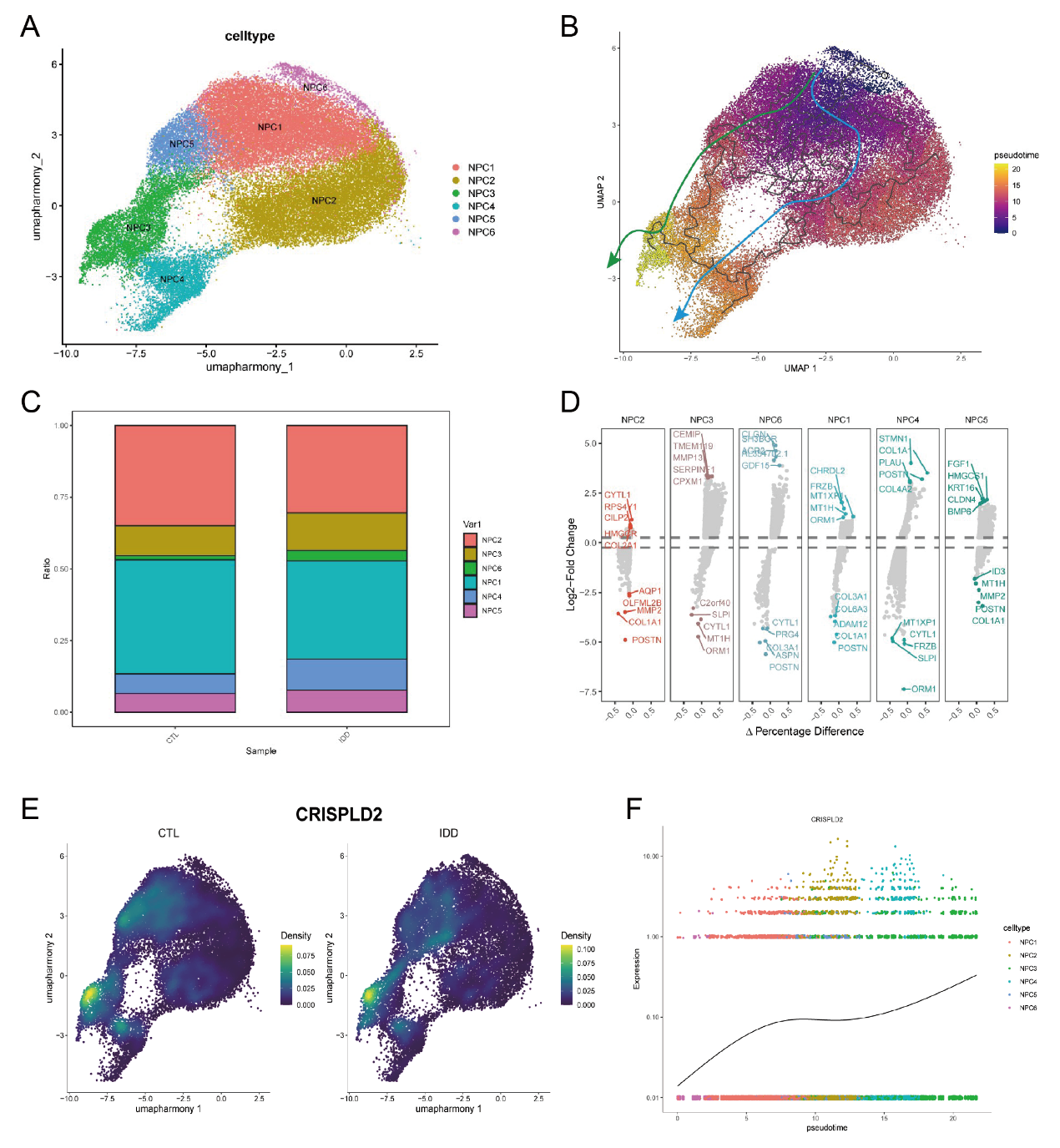


**Supplementary figure S2**. Pseudotime trajectory analysis reveals phenotypic transitions of NPCs and CRISPLD2 downregulation during IDD. (A) t-SNE plot showing six transcriptionally distinct NPC subtypes. (B) Pseudotime trajectory analysis indicates a continuum of cell states progressing from early-stage progenitor-like NPCs to late-stage dysfunctional phenotypes. (C) Bar graph illustrating proportional shifts in NPC subtypes between CTL and IDD samples, with a decrease in NPC1 and NPC2 and increase in NPC3-NPC6 in IDD. (D) Volcano plots showing differential expression of genes across NPC subtypes, highlighting high COL2A1 in NPC2 and elevated COL1A1/MMP13 in NPC3 and NPC4. (E) CRISPLD2 expression plotted on UMAP in CTL and IDD groups, demonstrating reduced expression in NPCs from degenerated discs.


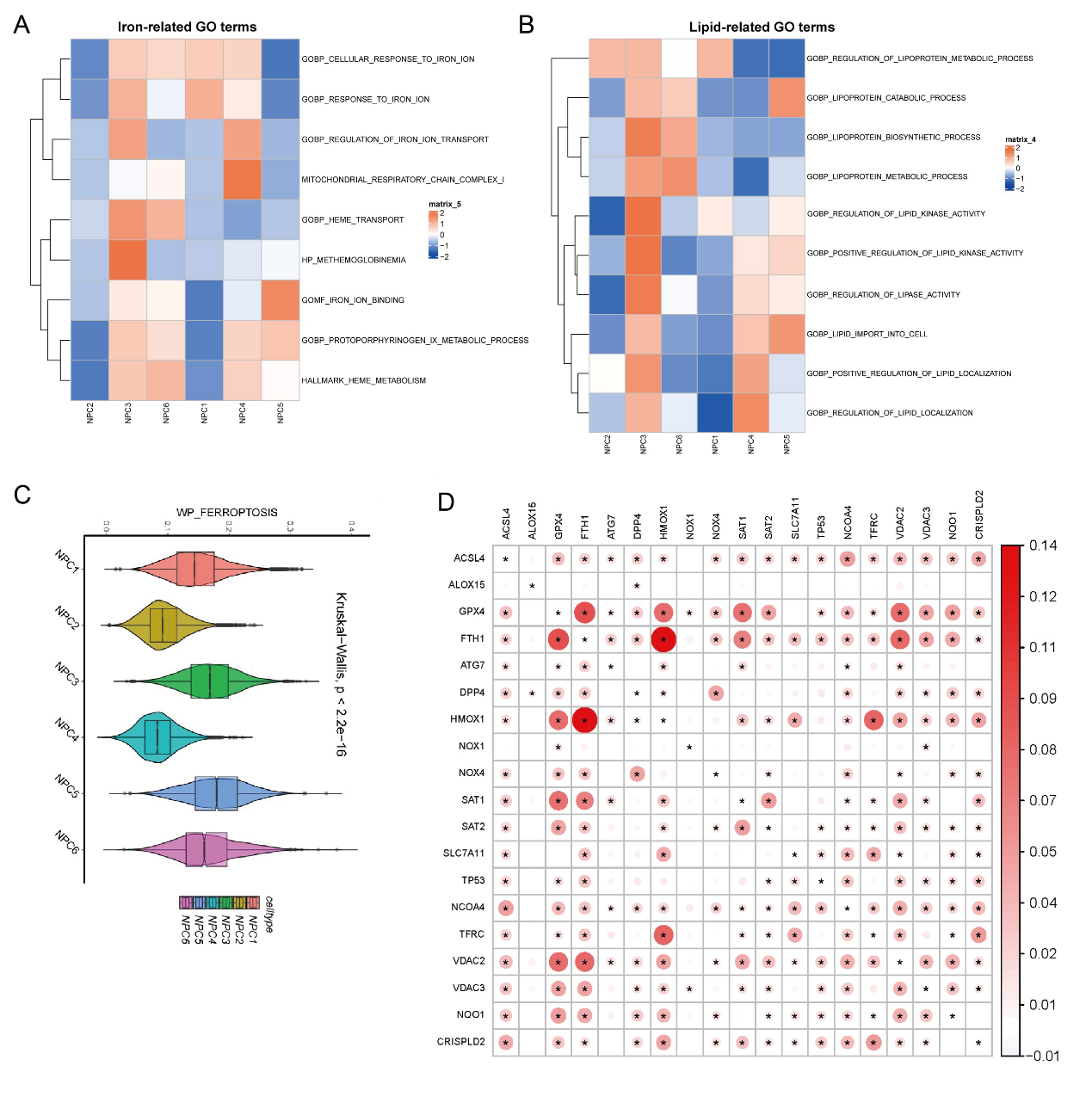


**Supplementary figure S3**. Transcriptomic clustering and ferroptosis-associated metabolic dysregulation in NPC subtypes during IDD. (A) Heatmap of iron metabolism-related GO terms enriched in NPC3, indicating disrupted iron homeostasis in degenerative subtypes. (B) Heatmap of lipid metabolism-related GO terms enriched in the same degenerative NPC subtype. (C) Violin plots of WP_FERROPTOSIS pathway activity across NPC subtypes. (D) Correlation matrix displaying interactions between CRISPLD2 and key ferroptosis-related genes.


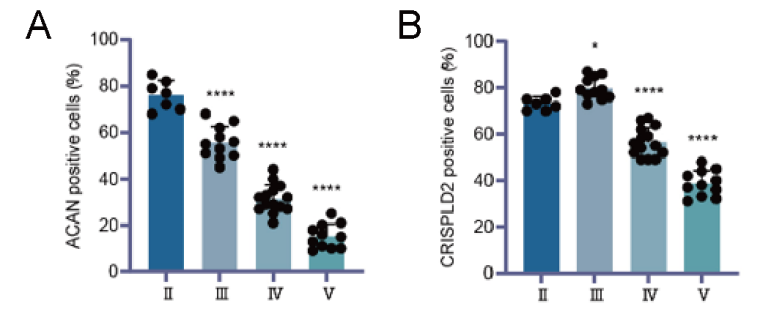


**Supplementary figure S4**. Quantification of immunohistochemical and immunofluorescence staining. (A) Quantification of ACAN positive cells. (B) Quantification of IF intensity of CRISPLD2. Data are presented as mean ±SD. Statistical analysis was performed using one-way ANOVA followed by Tukey’s post hoc test. **P* < 0.05, ***P* < 0.01, ****P* < 0.001, *****P* < 0.0001.


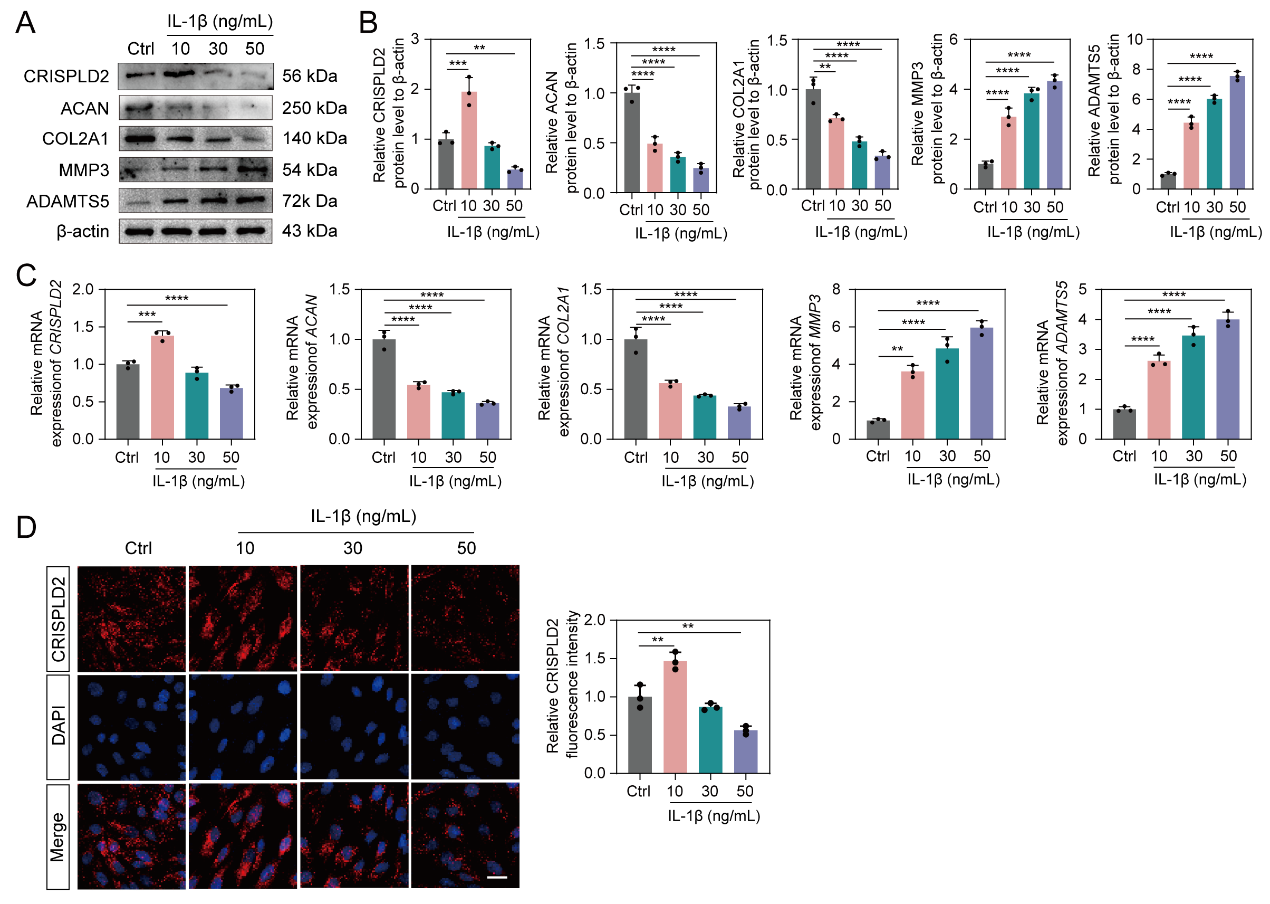


**Supplementary figure S5**. Time-dependent modulation of CRISPLD2 and matrix-related genes in NPCs following IL-1β stimulation. (A) Western blot analysis of CRISPLD2, ACAN, COL2A1, MMP3, and ADAMTS5 expression in NPCs treated with IL-1β (10 ng/mL) for 24, 48, and 72 hours. β-actin was used as a loading control. (B) Quantitative analysis of protein expression levels normalized to β-actin. (C) RT-RT-qPCR analysis of mRNA expression levels of *CRISPLD2*, *ACAN*, *COL2A1*, *MMP3*, and *ADAMTS5* under the same time-course conditions. (D) Immunofluorescence staining and quantification of CRISPLD2 in NPCs treated with IL-1β. Scale bar: 50 μm. Data represent mean ± SD (*n* = 3). Statistical comparisons were conducted using one-way ANOVA followed by Tukey’s post hoc test. **P* < 0.05, ***P* < 0.01, ****P* < 0.001, *****P* < 0.0001, ns = not significant. Scale bar, 50 μm.


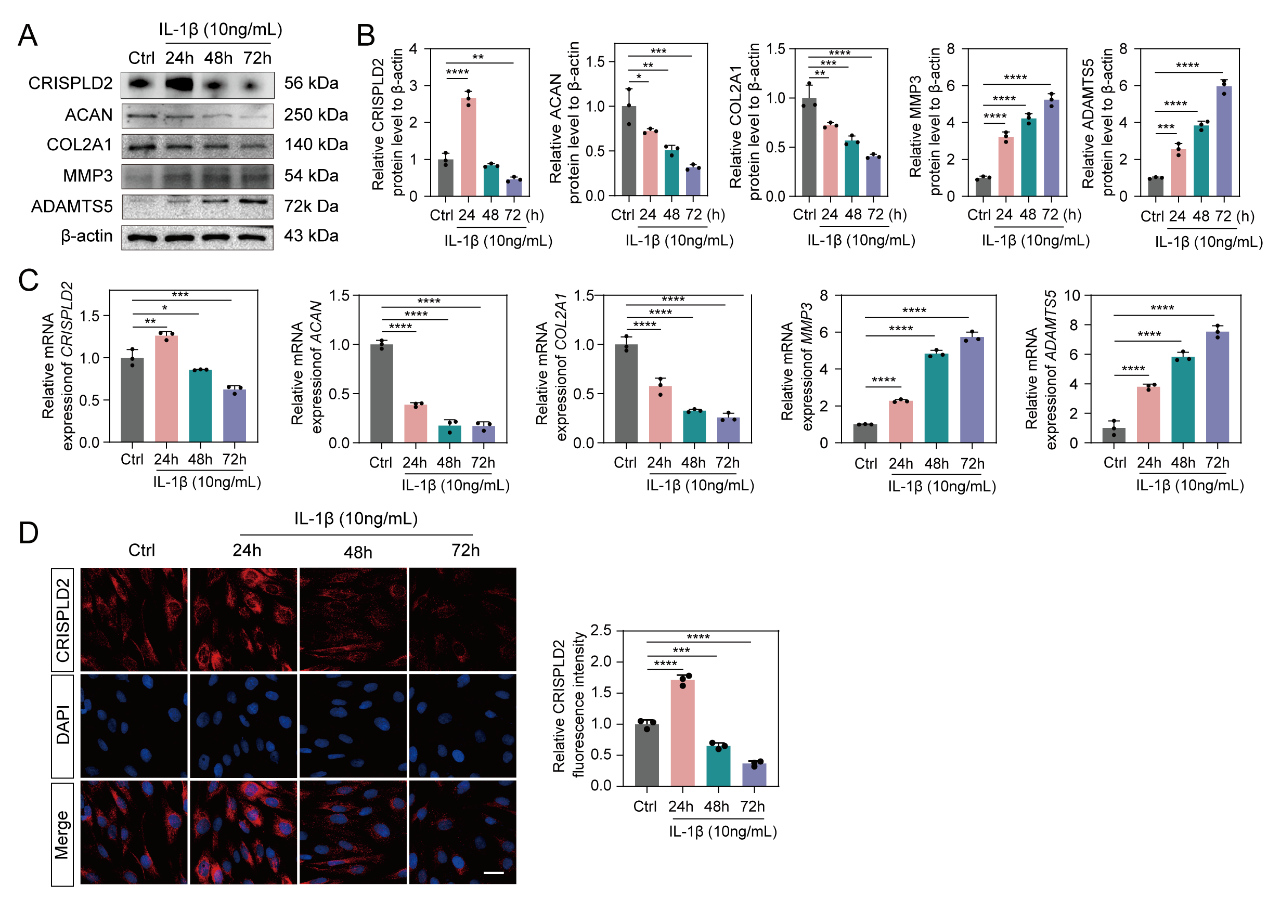


**Supplementary figure S6**. Dose-dependent effects of IL-1β on CRISPLD2 expression and extracellular matrix remodeling in NPCs. (A) Western blot analysis of CRISPLD2, ACAN, COL2A1, MMP3, and ADAMTS5 protein expression in NPCs treated with increasing concentrations of IL-1β (10, 30, and 50 ng/mL) for 24 hours. β-actin served as a loading control. (B) Quantitative analysis of protein expression normalized to β-actin. (C) RT-RT-qPCR analysis showing mRNA expression levels of *CRISPLD2*, *ACAN*, *COL2A1*, *MMP3*, and *ADAMTS5* under the same conditions. (D) Representative immunofluorescence images of CRISPLD2 in NPCs treated with IL-1β, and quantitative analysis of fluorescence intensity. Scale bar: 50 μm. Data are presented as mean ± SD (*n* = 3). Statistical comparisons were performed using one-way ANOVA followed by Tukey’s post hoc test. **P* < 0.05, ***P* < 0.01, ****P* < 0.001, *****P* < 0.0001, ns = not significant. Scale bar, 50 μm.


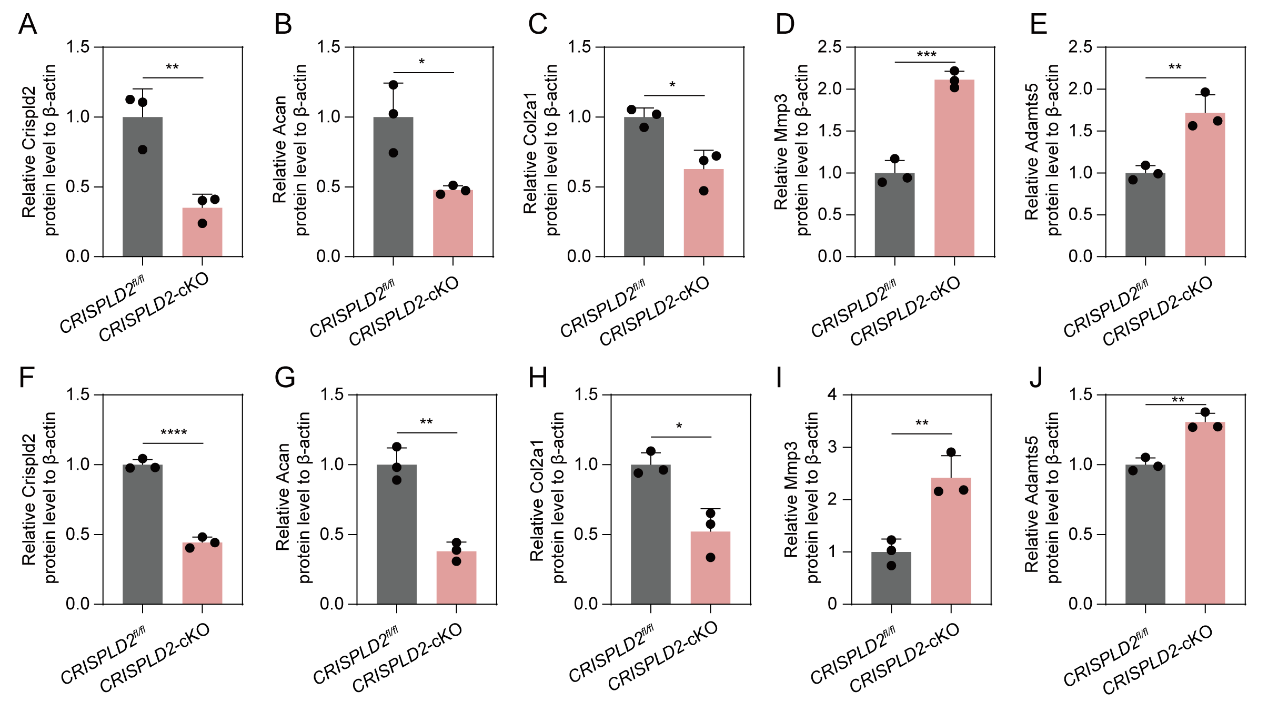


**Supplementary figure S7**. Quantitative analysis of ECM-related protein expression in lumbar and tail NP tissues from the *Crispld2^fl/fl^* and *Crispld2*-cKO mice. (A–E) Relative protein levels of Crispld2, Acan, Col2a1, Mmp3, and Adamts5 in lumbar NP tissues of the *Crispld2^fl/fl^* and *Crispld2*-cKO mice, normalized to β-actin. (F–J) Corresponding protein quantification in tail NP tissues. Data are presented as mean ± SD (*n* = 3). Statistical analysis was performed using unpaired two-tailed Student’s t test. **P* < 0.05, ***P* < 0.01, ****P* < 0.001, *****P* < 0.0001.


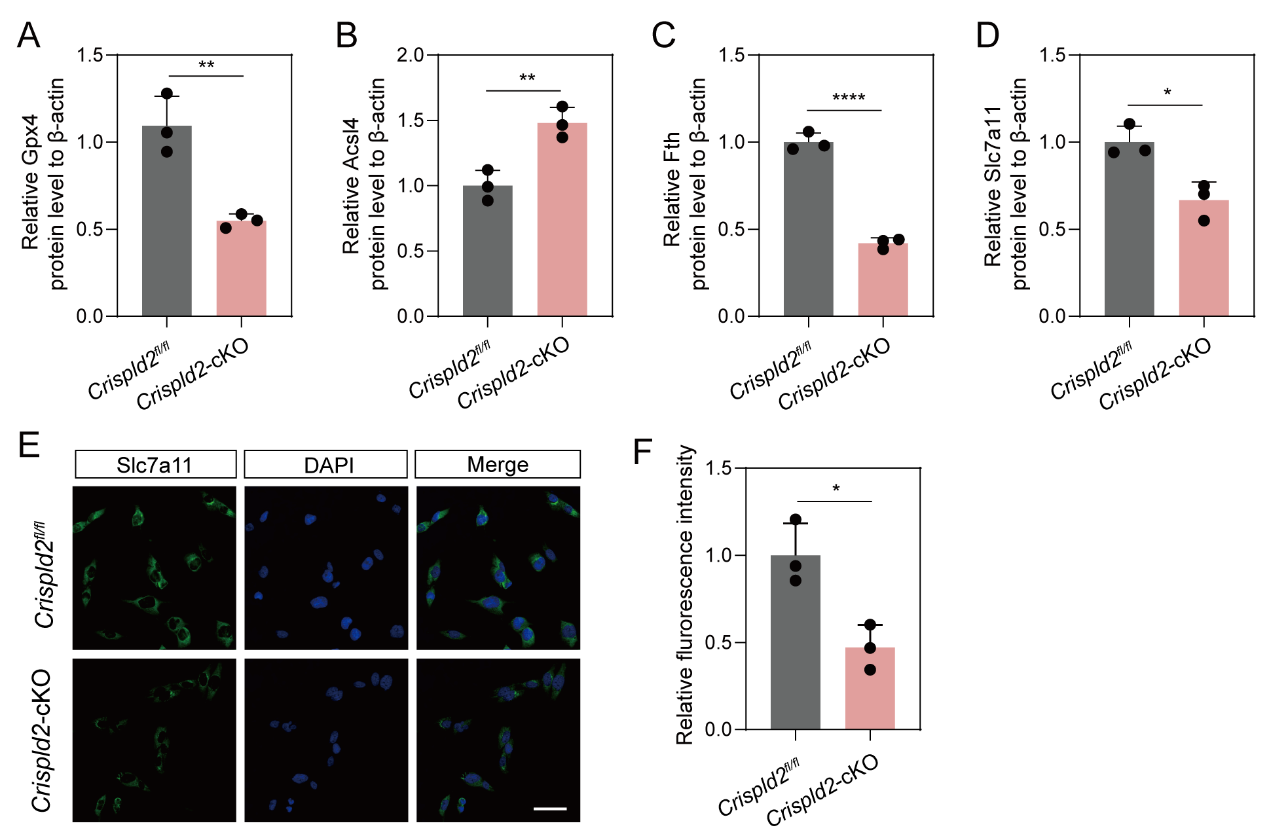


**Supplementary figure S8**. Quantifications of western blot and representative immunofluorescence images of Slc7a1. (A) Quantification of Gpx4. (B) Quantification of Acsl4. (C) Quantification of Fth. (D) Quantification of Slc7a11. (E and F) Representative immunofluorescence images and quantification of Slc7a1. Data are presented as mean ± SD (*n* = 3). Statistical comparisons were performed using two-tailed unpaired Student’s t test. **P* < 0.05, ***P* < 0.01, ****P* < 0.001, *****P* < 0.0001, ns = not significant.

**
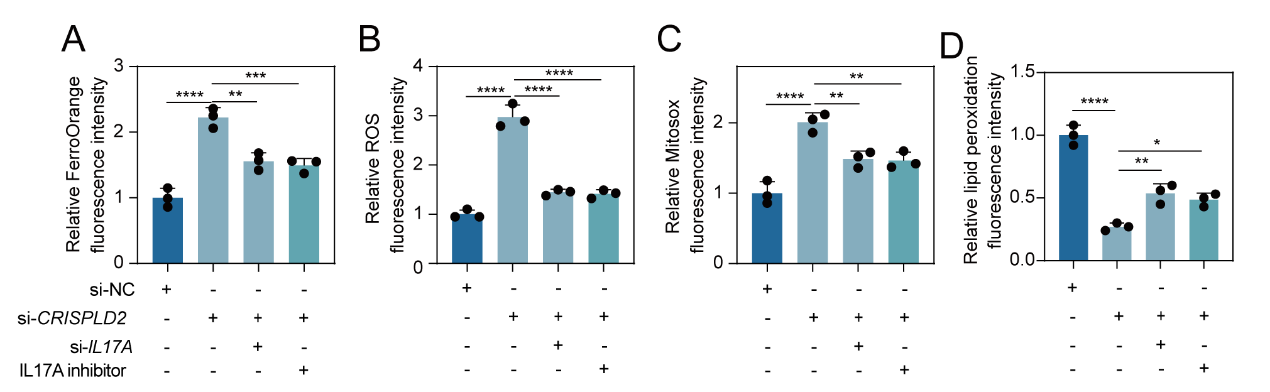
Supplementary figure S9**. Quantifications analysis of ferroptosis-related indicators. (A) Quantifications analysis of FerroOrange. (B) Quantifications analysis of ROS. (C) Quantifications analysis of MitoSOX. (D) Quantifications analysis of lipid peroxidation. Data are presented as mean ±SD (*n* = 3). Statistical comparisons were performed using one-way ANOVA followed by Tukey’s post hoc test. **P* < 0.05, ***P* < 0.01, ****P* < 0.001, *****P* < 0.0001, ns = not significant.
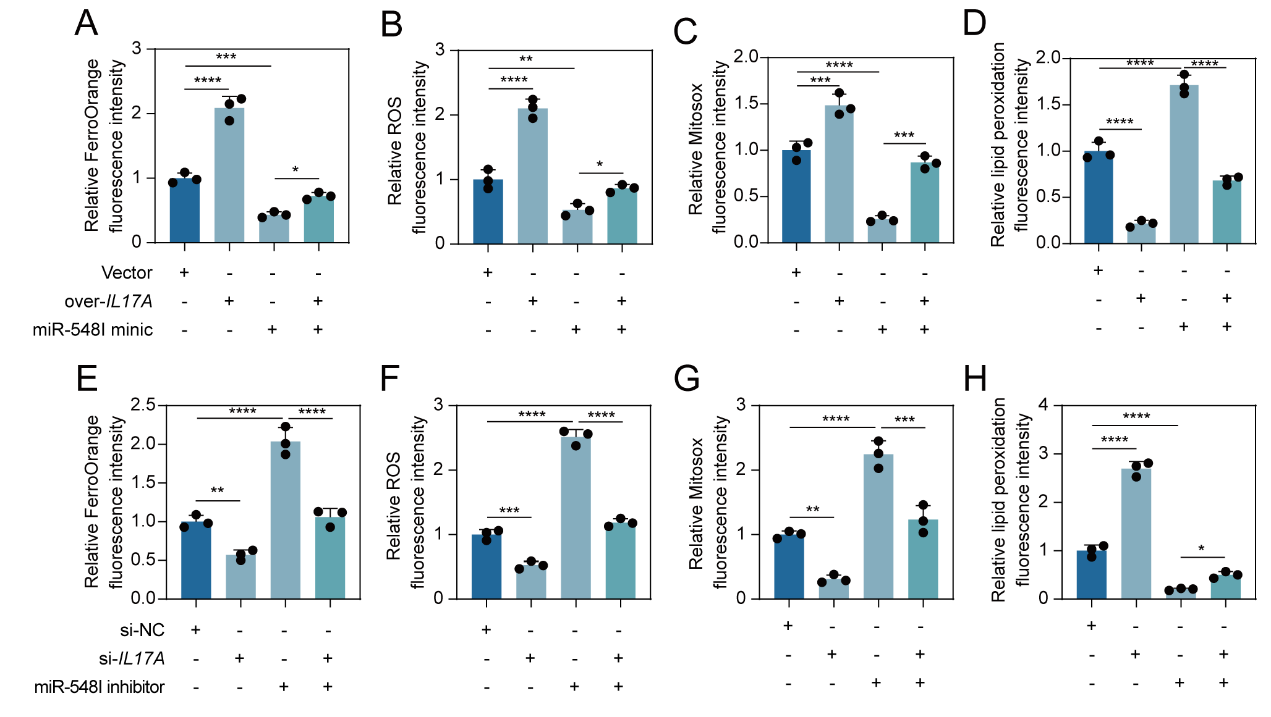


**Supplementary figure S10**. MiR-548i mitigates IL-17A-induced ferroptosis in NPCs. (A–D) Quantitative analysis of fluorescence intensity for FerroOrange, total ROS, mitochondrial ROS (MitoSOX), and lipid peroxidation in NPCs transfected with vector, overexpressed *IL-17A* (over-*IL17A*), miR-548i mimic, or over-*IL17A* + miR-548i mimic (*n* = 3). (E–H) Quantitative analysis of FerroOrange, total ROS, MitoSOX, and lipid peroxidation fluorescence in NPCs treated with si-NC, si-*IL17A*, miR-548i inhibitor, or si-*IL17A* + miR-548i inhibitor (*n* = 3). Data are presented as mean ± SD. Statistical comparisons were performed using one-way ANOVA followed by Tukey’s post hoc test. **P* < 0.05, ***P* < 0.01, ****P* < 0.001, *****P* < 0.0001, ns = not significant.


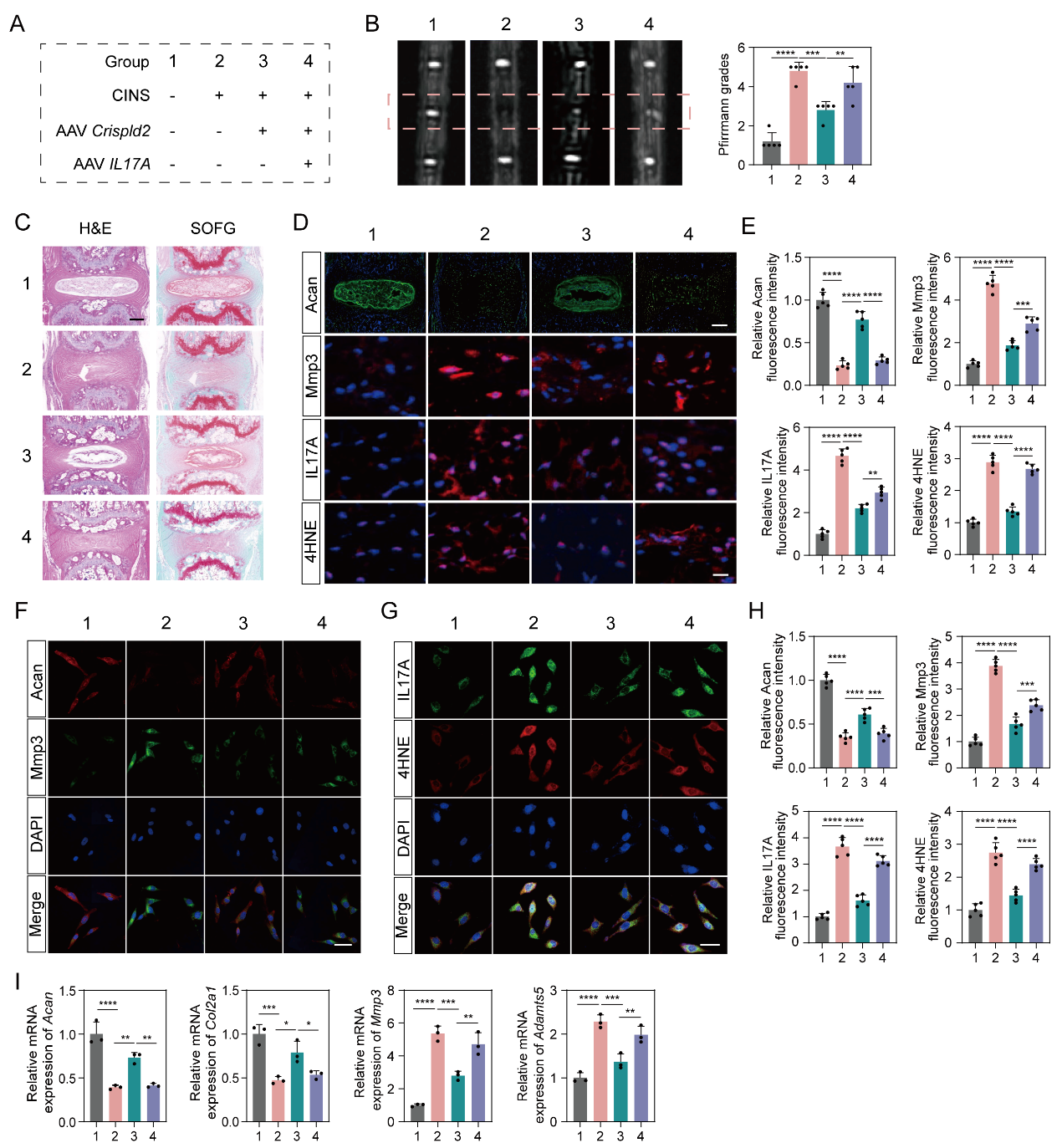
**Supplementary figure S11**. AAV- *Crispld2* protects against disc degeneration in the AFP model, reversed by IL-17A overexpression. (A) Experimental design for AFP mouse model: group 1 (control), group 2 (CINS), group 3 (AFP + AAV- *Crispld2*), group 4 (AFP + AAV- *Crispld2* + AAV-*IL17A*). (B) Representative MRI images and quantification of Pfirrmann grading score, (*n* = 5). (C) H&E and SOFG staining confirmed that AAV- *Crispld2* preserved disc integrity (*n* = 5). (D) Immunofluorescence staining of disc sections for Acan, Mmp3, IL17A, and 4HNE. Scale bar: 500 μm (Acna), 50 μm (Mmp3, IL17A, and 4HNE) (*n* = 5). (E) Quantification of protein and miRNA fluorescence intensities (*n* = 5). (F–G) Immunofluorescence staining of isolated NPCs. Scale bar: 50 μm (*n* = 5). (H) Quantification of protein levels in NPCs (*n* = 5). (I) RT-qPCR analysis for disc matrix and catabolic markers in NP tissues (*n* = 3). Data are presented as mean ± SD. Comparisons were performed using one-way ANOVA with Tukey’s post hoc test. **P* < 0.05, ***P* < 0.01, ****P* < 0.001, *****P* < 0.0001, ns = not significant.


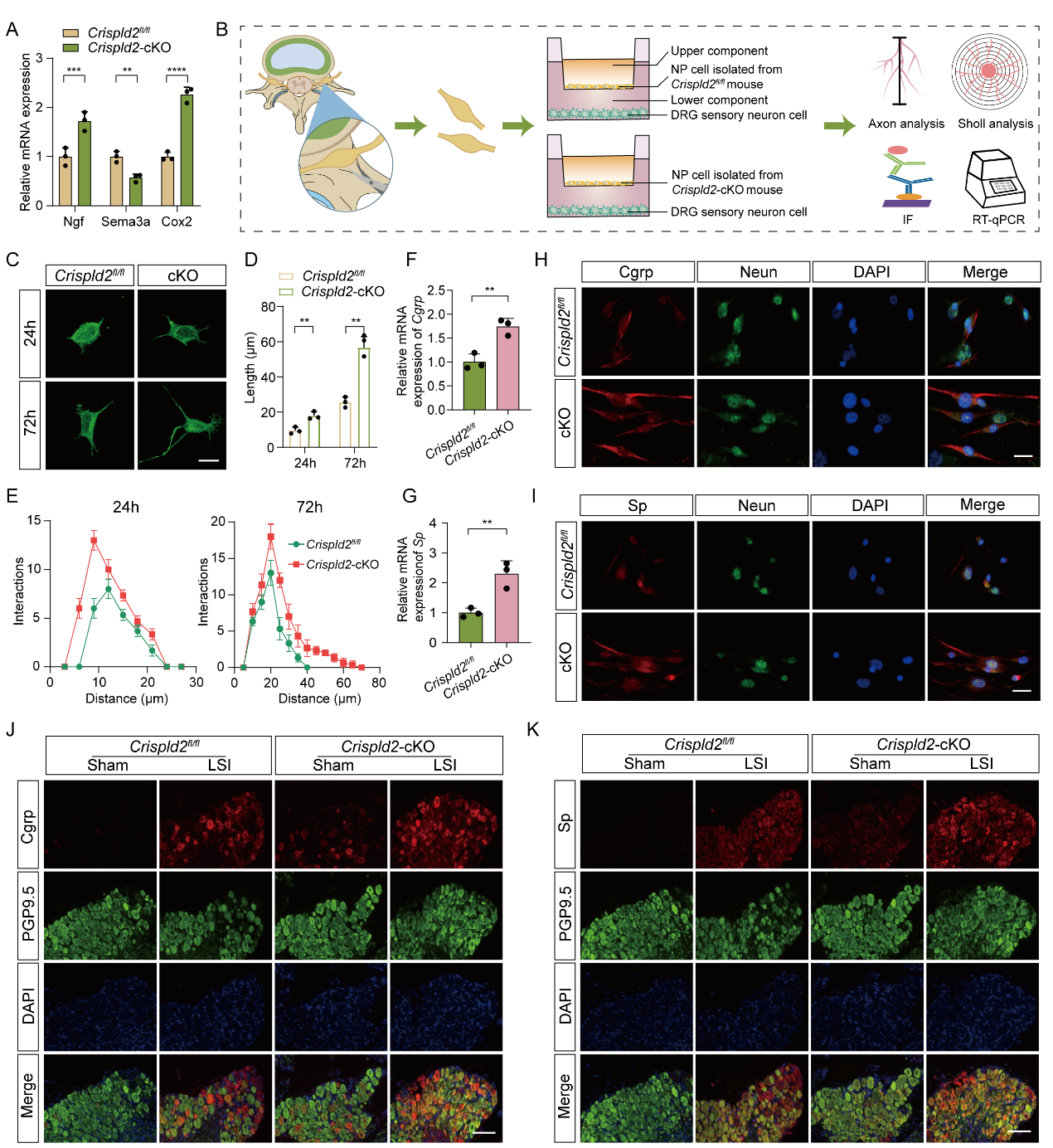


**Supplementary figure S12**. *Crispld2* deficiency induces discogenic pain via hyperalgesia and enhanced nociceptive signaling. (A) RT-qPCR analysis of pain- and nerve growth-related markers in NPCs following *Crispld2* knockdown (*n* = 3). (B) Schematic diagram illustrating the experimental setup of an indirect co-culture system between *Crispld2*-deficiency NPCs and DRG neurons. (C) Representative immunofluorescence images of DRG neurons stained with NF200 after co-culture with NPCs from *Crispld2^fl/fl^* and *Crispld2*-cKO mice for 24 and 72 hours. Scale bar: 20 μm (*n* = 3). (D) Quantification of neurite length in DRG neurons at 24 h and 72 h (*n* = 3). (E) Sholl analysis revealed increased neurite branching in DRG neurons exposed to *Crispld2*-deficiency NPCs at both time points (*n* = 3). (F–G) RT-qPCR showing upregulation of *Cgrp* and *Sp* expression in DRG neurons co-cultured with *Crispld2*-deficient NPCs (*n* = 3). (H–I) Representative immunofluorescence staining for Cgrp and Sp in DRG neurons co-cultured with NPC from *Crispld2^fl/fl^* and *Crispld2*-cKO mice. Scale bar: 20 μm (*n* = 3). (J–K) Immunofluorescence staining of lumbar DRG sections from *Crispld2^fl/fl^* and *Crispld2*-cKO mice, probed with Cgrp, Sp, and neuronal marker PGP9.5. Scale bar: 100 μm (*n* = 5). Data represent mean ± SD. Comparisons were performed by unpaired Student’s t-test. **P* < 0.05, ***P* < 0.01, ****P* < 0.001, *****P* < 0.0001, ns = not significant.

**
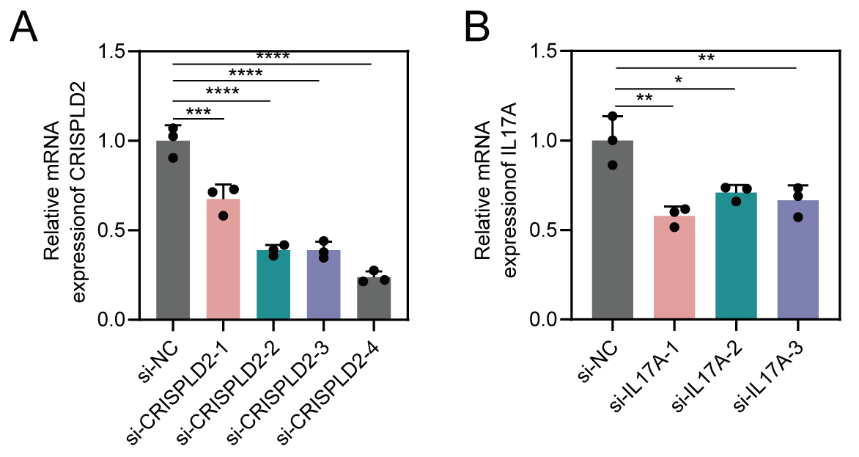
**

**Supplementary figure S13**. The results of RT-RT-qPCR for the selection of the best siRNA for human *CRISPLD2* (A) and *IL17A* (B)*.* **P < 0.05, **P < 0.01, ***P < 0.001, ****P < 0.0001*

Table S1 Demographic data of patients

| Patient no | Age | Gender | Level | Pfirrmann grading |
| --- | --- | --- | --- | --- |
| 1 | 62 | M | L2-L3 | Ⅳ |
| 2 | 58 | M | L4-L5 | Ⅲ |
| 3 | 21 | M | L3-L4 | Ⅱ |
| 4 | 49 | M | L5-S1 | Ⅳ |
| 5 | 71 | M | L4-L5 | Ⅴ |
| 6 | 52 | M | L2-L3 | Ⅲ |
| 7 | 42 | M | L4-L5 | Ⅳ |
| 8 | 35 | M | L3-L4 | Ⅱ |
| 9 | 64 | M | L5-S1 | Ⅳ |
| 10 | 68 | M | L4-L5 | Ⅳ |
| 11 | 53 | M | L2-L3 | Ⅲ |
| 12 | 57 | M | L5-S1 | Ⅴ |
| 13 | 39 | M | L2-L3 | Ⅲ |
| 14 | 48 | M | L3-L4 | Ⅴ |
| 15 | 29 | M | L5-S1 | Ⅱ |
| 16 | 37 | M | L4-L5 | Ⅲ |
| 17 | 63 | M | L2-L3 | Ⅳ |
| 18 | 43 | M | L5-S1 | Ⅲ |
| 19 | 73 | M | L5-S1 | Ⅴ |
| 20 | 34 | M | L4-L5 | Ⅱ |
| 21 | 52 | M | L5-S1 | Ⅳ |
| 22 | 49 | F | L2-L3 | Ⅲ |
| 23 | 37 | F | L5-S1 | Ⅳ |
| 24 | 51 | F | L4-L5 | Ⅴ |
| 25 | 26 | F | L3-L4 | Ⅱ |
| 26 | 29 | F | L5-S1 | Ⅲ |
| 27 | 38 | F | L3-L4 | Ⅱ |
| 28 | 63 | F | L2-L3 | Ⅴ |
| 29 | 34 | F | L3-L4 | Ⅱ |
| 30 | 42 | F | L4-L5 | Ⅳ |
| 31 | 34 | F | L3-L4 | Ⅴ |
| 32 | 48 | F | L4-L5 | Ⅲ |
| 33 | 67 | F | L3-L4 | Ⅳ |
| 34 | 70 | F | L2-L3 | Ⅳ |
| 35 | 60 | F | L3-L4 | Ⅴ |
| 36 | 46 | F | L3-L4 | Ⅳ |
| 37 | 57 | F | L5-S1 | Ⅳ |
| 38 | 63 | F | L4-L5 | Ⅴ |
| 39 | 54 | F | L3-L4 | Ⅴ |
| 40 | 41 | F | L4-L5 | Ⅲ |
| 41 | 62 | F | L3-L4 | Ⅴ |
| 42 | 53 | F | L4-L5 | Ⅳ |
| 43 | 66 | F | L4-L5 | Ⅲ |

Table S2 Primer sequences for mRNA detection

| Species | Gene | Primer type | Sequence |
| --- | --- | --- | --- |
| Homo | *CRISPLD2* | F | ACAGTCCGTCCCTACCAATAG |
|  |  | R | AGCCCTCTTACTCTAGCCCAT |
| Homo | *ACAN* | F | TGGAGACAAGGATGAGTTTCC |
|  |  | R | GGCGAAGCAGTACACATCATA |
| Homo | *COL2A1* | F | CCAGAAACAACACAATCCGTT |
|  |  | R | ATGGACATCAGGTCAGGTCAG |
| Homo | *MMP3* | F | AACTTGAGCGTGAATCTGTATC |
|  |  | R | TGTGACAAGGTGCAAGCTA |
| Homo | *ADAMTS* | F | CTGTGACCAAAAGAGGATGTG |
|  |  | R | AGTGTTTCTTGTAAGCCCAGG |
| Homo | *ACTB* | F | AAGGTGACAGCAGTCGGTT |
|  |  | R | TGTGTGGACTTGGGAGAGG |
| Mouse | *Crispld2* | F | GCTGCTATCCACTACGGTGTC |
|  |  | R | AGACGGCTTGTATTTGCTCAG |
| Mouse | *Acan* | F | GTTAGTGGAGGGTGTGACTGAA |
|  |  | R | CGGGGAGTGTATGTCATAGAAG |
| Mouse | *Col2al* | F | GCTCCCCTTTCTAAGAGACC |
|  |  | R | TGACTCACACCAGATAGTTCC |
| Mouse | *Mmp3* | F | GGCATCCTGTGTTTTAACTGA |
|  |  | R | CCATAGCTCCTGTTTGGTTCT |
| Mouse | *Adamts5* | F | CTTCAATCCTTACCAGCATCG |
|  |  | R | TTACCATGACCATCATCCAGG |
| Mouse | *Cgrp* | F | GGCGAAGCAGTACACATCATA |
|  |  | R | CCAGAAACAACACAATCCGTT |
| Mouse | *Sp* | F | GGCGAAGCAGTACACATCATA |
|  |  | R | CCAGAAACAACACAATCCGTT |
| Mouse | *Sema3a* | F | CCAGCAGGCTTTCTTCAGTAT |
|  |  | R | GGAAACACTAAGTCAGCGGAC |
| Mouse | *Ngf* | F | TACAGGCAGAACCGTACACAG |
|  |  | R | GTGTCAAGGGAATGCTGAAGT |
| Mouse | *Cox2* | F | GTTAGTGGAGGGTGTGACTGAA |
|  |  | R | CGGGGAGTGTATGTCATAGAAG |

Table S3 The synthetic siRNA sequences for human *CRISPLD2* and *IL17A*

| Source | Gene name | Sequences |
| --- | --- | --- |
| GenePharma | *CRISPLD2*-si-1 | 5’- CAGUCCCUCAGCAAAUACATT -3’  5’- UGUAUUUGCUGAGGGACUGTT -3’ |
|  | *CRISPLD2*-si-2 | 5’- CCGAGAAGAAACCUACACUTT -3’  5’- AGUGUAGGUUUCUUCUCGGTT -3’ |
|  | *CRISPLD2*-si-3 | 5’- GUCUGCAAUUAUUCUCCAATT -3’  5’- UUGGAGAAUAAUUGCAGACTT -3’ |
|  | *CRISPLD2*-si-4 | 5’- CAAGUCGUCAGAUGUGACATT -3’  5’- UGUCACAUCUGACGACUUGTT -3’ |
| Genomeditech | *IL17A*-si-1 | 5’-GGUCCUCAGAUUACUACAACC-3’  5’-UUGUAGUAAUCUGAGGACCUU-3’ |
|  | *IL17A*-si-2 | 5’-CGUGGACUACCACAUGAACUC-3’  5’-GUUCAUGUGGUAGUCCACGUU-3’ |
|  | *IL17A*-si-3 | 5’-GAGCUAUUUAAGGAUCUAUUUTT-3’  5’-AAAUAGAUCCUUAAAUAGCUCTT-3’ |
